# Causes of death among patients with hepatocellular carcinoma in United States from 2000 to 2018

**DOI:** 10.1002/cam4.5986

**Published:** 2023-04-21

**Authors:** Zhen Yang, Kaiming Leng, Guangjun Shi

**Affiliations:** ^1^ Department of Hepatopancreatobiliary Surgery, Qingdao Municipal Hospital Qingdao University Qingdao People's Republic of China; ^2^ Department of Hepatopancreatobiliary Surgery, Qingdao Hospital University of Health and Rehabilitation Sciences (Qingdao Municipal Hospital) Qingdao People's Republic of China

**Keywords:** cancer survivorship, causes of death, hepatocellular carcinoma, SEER

## Abstract

**Background:**

The gains in survival outcomes of US patients with hepatocellular carcinoma (HCC) have come at the expense of developing non‐cancer‐related morbidities, such as cardiovascular diseases (CVDs) and infections. However, population‐based data on causes of death (CODs) in patients with HCC are scarce.

**Methods:**

A cancer registry database in the United States was used to analyze the CODs among patients with HCC. Death cause distribution and standardized mortality ratios were calculated to quantify the disease‐specific death burden.

**Results:**

A total of 40,094 patients with a histological diagnosis of HCC were identified from the SEER‐18 database between 2000 and 2018, of which 30,796 (76.8%) died during the follow‐up period. The majority of these deaths (25,153, 81.7%) occurred within 2 years after diagnosis, 13.2% (4075) occurred within 2–5 years, and 5.1% (1568) occurred after 5 years. All age groups had a lower burden of female deaths than of male deaths during the study period. With respect to CODs, 23,824 (77.4%), 2289 (7.4%), and 4683 (15.2%) were due to HCC, other cancers, and non‐cancer causes, respectively. Non‐cancer‐related deaths were more common among older patients and those with longer latency periods since diagnosis. The major causes of non‐cancer‐related deaths are other infectious and parasitic diseases, including HIV and CVDs.

**Conclusions:**

CODs during HCC survivorship varied, and a growing number of survivors tended to die from causes other than HCC, with an increasing latency period since diagnosis. Comprehensive analyses of mortality patterns and temporal trends could underpin strategies to reduce these risks.

## 1 INTRODUCTION

Liver cancer, one of the most common fatal malignancies, is the fifth leading cause of cancer‐related deaths in the United States (US), with an estimated 100,000 patients in 2040.^1^, ^2^, ^3^ Hepatocellular carcinoma (HCC), which arises from hepatocytes, accounts for nearly 90% of all liver cancer cases.^4^, ^5^ As one of the most frequently diagnosed diseases worldwide, the number of survivors of HCC is also rapidly growing because of considerable progress in oncology practices and outcomes.^6^, ^7^ Five‐year overall survival rate among US patients with HCC increased from 11.7% in 2000 to 21.3% in 2011.^8^ Unfortunately, gains in survival outcomes have come at the cost of long‐term morbidities such as cardiovascular diseases (CVDs) and infections.^9^, ^10^, ^11^ A large body of evidence has substantiated the heightened burden of CVDs among patients after a diagnosis of cancer compared with cancer‐free persons.^12^, ^13^, ^14^ This spike in CVDs risk is largely a consequence of age‐related pathologies coupled with the adverse effects of cancer treatments.^15^, ^16^ The risk of death from CVDs is prone to change with the evolution of management strategies aimed at cancer and cardiovascular conditions. Similarly, the increased risk of infections among cancer patients could be interpreted in part to be the result of treatment as well as the burden of living with a progressing tumor.^17^, ^18^ All these comorbid illnesses are negatively associated with prognosis and have become a major public health concern worldwide.

Considering that many patients would survive their disease for several years after cancer diagnosis, identifying the causes of death (CODs) in these populations could confer survival benefits and assist in multidisciplinary treatment planning. Furthermore, a better understanding of the actual causes and changes in the risk of death among contemporary HCC survivors could help with proper healthcare prioritization during survivorship. However, few studies have examined CODs among patients with HCC in the United States, particularly based on population‐based data.

In this study, we aimed to characterize primary CODs and improve the understanding of their distribution among patients after HCC diagnosis, with the goal of providing effective and viable management strategies to mitigate the risk of death.

## 2 MATERIALS AND METHODS

### 2.1 Study design

A large retrospective cohort study based on the National Cancer Institute's Surveillance, Epidemiology, and End Results (SEER)‐18 registries was conducted to determine CODs in patients who were histologically diagnosed with HCC (HCC cases were identified using anatomic site [liver, C22.0] and morphology codes [8170–8175] according to the International Classification of Disease for Oncology, third edition [ICD‐O‐3]).

### 2.2 Study population

Individuals with a histological diagnosis of HCC as their first malignant neoplasm were abstracted from the SEER‐18 database during 2000 and 2018, which covering about 28% of the US population. Information on patient demographics, characteristics, treatment, CODs, and vital status was collected and analyzed. To minimize the risk of selection bias, our study included all eligible HCC cases documented in the SEER‐18 registry. Cases without positive histology or complete data on disease staging were not included in our study. Patients who were diagnosed with HCC through a death certificate or autopsy only, as well as those with unknown vital status, were excluded from the analysis. Written informed consent was not required as the SEER data were anonymized and publicly available.

### 2.3 Outcome assessments

CODs among patients with HCC was inspected according to the following variables: sex (female/male), ethnicity (White/Black/American Indian or Alaska Native [AIAN]/Asian or Pacific Islander [API]), age at diagnosis (<50/50–65 and >65 years), cancer stage (localized/regional/distant), and treatment (surgery/chemotherapy/radiation), each stratified by the latency period after HCC diagnosis (<2, 2–5, and >5 years). CODs were categorized by the ICD‐9 code, and the underlying causes were coded as “HCC,” “other cancers,” and “non‐cancer death” (i.e., deaths attributed to any cause other than cancer). Examples of non‐cancer deaths include CVDs, which include heart disease, hypertension, cerebrovascular disease, and arterial diseases. Infectious diseases in our study were classified as pneumonia, influenza, septicemia, and other infectious and parasitic diseases, including HIV. Other non‐cancer causes include chronic obstructive pulmonary disease (COPD), nephritis, nephrotic syndrome and nephrosis, accidents, and adverse effects, suicide, and self‐inflicted injuries. The proportions of disease‐specific deaths among patients with HCC within each time period and age group were further analyzed in our study.

### 2.4 Statistical analyses

Standardized mortality ratios (SMRs), defined as the observed‐to‐expected number of deaths, were calculated to estimate the relative risks of cause‐specific mortality compared to the general US population. The expected number of deaths was calculated for a population demographically similar to the study population over the same period, after adjusting for age, sex, and race/ethnicity, by multiplying the general population mortality rates for each cause of death by person‐years at risk. The mortality rates for the general population were collected between 1969 and 2018 and retrieved using SEER*Stat software, specifically from “Mortality‐All COD, Aggregated Total U.S. (1969–2018) <Katrina/Rita Population Adjustment>.” The SMRs in the current study represent the relative risk of death after a histological diagnosis of HCC in comparison with the risk of the reference population in the United States. The SMRs and corresponding 95% confidence intervals (CIs) were calculated using the exact method in the SEER*Stat software, version 8.3.6. All statistical tests were two‐sided, and a *p*‐value <0.05 was deemed as having statistically significant.

## 3 RESULTS

### 3.1 Baseline characteristics

**FIGURE 1:**
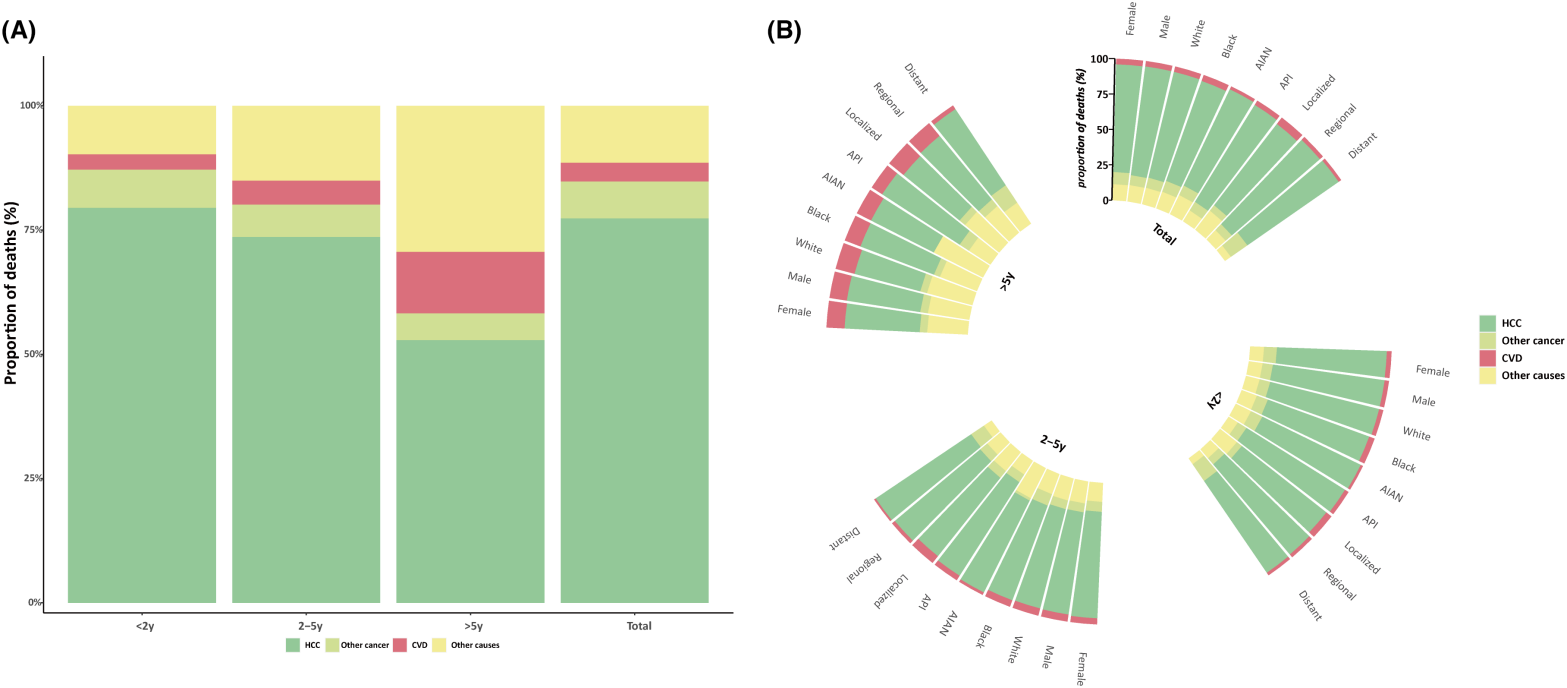
Causes of death during each latency period (<2 years, 2–5 years, and >5 years) after HCC diagnosis. AIAN, American Indian or Alaska Native; API, Asian or Pacific Islander; CVD, cardiovascular disease; HCC, hepatocellular carcinoma.

A total of 40,094 patients with a histological diagnosis of HCC were identified from the SEER‐18 database between 2000 and 2018, of which 30,796 (76.8%) died during the follow‐up period. The mean age at death was 63.04 years. Of all eligible patients, the vast majority were older than 50 years (90.0%), men (77.2%), and white (67.2%). Almost 80% of patients were diagnosed with local‐regional diseases. However, only one‐third of patients underwent surgical treatment and adjuvant chemotherapy (Table 1). Of the total deaths, most (25,153, 81.7%) occurred within 2 years after cancer diagnosis, 13.2% (4075) died between 2 and 5 years, and 5.1% (1568) died after 5 years. With respect to CODs, 23,824 (77.4%), 2289 (7.4%), and 4683 (15.2%) deaths were due to HCC, other cancers, and non‐cancer causes, respectively (Table 2). In the period of 2000–2018, the major causes of non‐cancer deaths were other infectious and parasitic diseases, including HIV (1214; SMR, 98.56; 95% CI, 101.85–215.65), and CVDs (1160; SMR, 2.28; 95% CI, 2.19–2.38). Figure 1 shows the time‐dependent proportion of cause‐specific deaths after HCC diagnosis. More patients were threatened with HCC‐related deaths within 5 years of diagnosis than thereafter (Figure 1A). As shown in Figure 1A, HCC accounted for the majority of CODs over the entire study period, whereas the proportion of CVDs and other causes significantly increased after 5 years after cancer diagnosis. Time‐dependent CODs stratified by key patient characteristics also suggested that HCC was the main COD in all subgroups, whereas the fraction of non‐HCC causes was significantly increased among those with longer latency after diagnosis (Figure 1B). Notably, CVD‐related mortality showed a slight increase among patients with localized stage disease as well as those exceeding 5 years after their cancer diagnosis.

**TABLE 1:** Baseline characteristics of patients with HCC and of those who died according to the time of death after diagnosis.

Characteristics	Diagnosed cases, no. (%)	Deaths, no. (%)	Age at death, mean (SD) (years)	Deaths by time after diagnosis, no. (%)		
				<2 years	2–5 years	>5 years
All patients	40,094 (100)	30,796 (100)	63.04	25,153 (81.7)	4075 (13.2)	1568 (5.1)
Age at diagnosis						
<50 years	4000 (10.0)	2867 (9.3)	41.46	2315 (9.2)	369 (9.1)	183 (11.6)
50–65 years	18,618 (46.4)	13,784 (44.8)	57.73	11,105 (44.1)	1902 (46.7)	777 (49.6)
≥65 years	17,476 (43.6)	14,145 (45.9)	73.64	11,733 (46.6)	1804 (44.3)	608 (38.8)
Gender						
Female	9155 (22.8)	6850 (22.2)	65.77	5549 (22.1)	924 (22.7)	377 (24.0)
Male	30,939 (77.2)	23,946 (77.8)	62.23	19,604 (77.9)	3151 (77.3)	1191 (76.0)
Ethnicity						
White	26,949 (67.2)	20,916 (67.9)	63.60	17,158 (68.3)	2721 (66.8)	1037 (66.2)
Black	5685 (14.2)	4637 (15.1)	60.36	3933 (15.6)	525 (12.9)	179 (11.4)
AIAN	485 (1.2)	378 (1.2)	62.08	305 (1.2)	55 (1.3)	18 (1.1)
API	6975 (17.4)	4865 (15.8)	63.13	3757 (14.9)	774 (19.0)	334 (21.3)
Tumor stage						
Localized	20,676 (51.6)	13,617 (44.2)	63.33	9539 (38.0)	2823 (69.3)	1255 (80.0)
Regional	11,364 (28.3)	9621 (31.2)	62.83	8339 (33.1)	1003 (24.6)	279 (17.8)
Distant	8054 (20.1)	7558 (24.5)	62.60	7275 (28.9)	249 (6.1)	34 (2.2)
Treatment						
Surgery	13,981 (34.9)	7439 (24.2)	60.70	4071 (16.2)	2147 (52.7)	1221 (77.9)
Chemotherapy	13,953 (34.8)	10,758 (34.9)	61.78	8451 (33.6)	1804 (44.3)	503 (32.1)
Radiation	3647 (8.6)	2762 (9.0)	63.93	2399 (9.5)	317 (7.8)	46 (2.9)

Abbreviations: AIAN, American Indian or Alaska Native; API, Asian or Pacific Islander; HCC, hepatocellular carcinoma; SD, standard deviation.

**TABLE 2:** Observed deaths and SMRs for causes of death after diagnosis of HCC.

Cause of death	Deaths by time after diagnosis						Total deaths	
	<2 years		2–5 years		>5 years			
	Observed, no.	SMR (95% CI)	Observed, no.	SMR (95% CI)	Observed, no.	SMR (95% CI)	Observed, no.	SMR (95% CI)
All	25,153	34.67* (34.38, 34.96)	4075	11.26* (11.03, 11.49)	1568	4.47* (4.32, 4.63)	30,796	21.74* (21.58, 21.91)
HCC	19,994	NA	3001	NA	829	NA	23,824	NA
Other cancers	1938	9.43* (9.14, 9.74)	266	3.39* (3.15, 3.65)	85	2.11* (1.90, 2.34)	2289	6.20* (6.02, 6.37)
Non‐cancer causes	3221	7.10* (6.94, 7.25)	808	3.26* (3.11, 3.40)	654	2.29* (2.15, 2.42)	4683	5.01* (4.92, 5.11)
Cardiovascular diseases	771	3.00* (2.85, 3.15)	195	1.55* (1.41, 1.71)	194	1.45* (1.29, 1.61)	1160	2.28* (2.19, 2.38)
Septicemia	119	10.75* (9.47, 12.16)	26	5.11* (3.93, 6.54)	22	3.32* (2.30, 4.64)	167	7.60* (6.83, 8.44)
Pneumonia and influenza	53	3.23* (2.66, 3.89)	23	2.39* (1.73, 3.23)	16	1.82* (1.21, 2.64)	92	2.69* (2.32, 3.11)
COPD	100	2.36* (2.06, 2.70)	26	1.14 (0.85, 1.48)	32	1.52* (1.17, 1.95)	158	1.85* (1.65, 2.06)
Other infectious and parasitic diseases including HIV	905	147.22* (141.82, 152.78)	205	57.20* (52.55, 62.15)	104	27.82* (24.15, 31.89)	1214	98.56* (101.85, 215.65)
Diabetes mellitus	110	4.26* (3.75, 4.83)	41	2.31* (1.81, 2.92)	32	2.09* (1.57, 2.73)	183	3.25* (2.92, 3.60)
Nephritis, nephrotic syndrome and nephrosis	89	6.12* (5.29, 7.04)	19	3.08* (2.29, 4.05)	42	4.41* (3.38, 5.67)	150	4.93* (4.39, 5.51)
Accidents and adverse effects of medications	104	4.53* (3.99, 5.12)	41	3.41* (2.78, 4.15)	35	2.35* (1.77, 3.05)	180	3.75* (3.39, 4.13)
Suicide and self‐inflicted injury	23	2.47* (1.78, 3.34)	7	1.67 (0.93, 2.76)	5	1.27 (0.58, 2.42)	35	2.00* (1.55, 2.54)
Other	947	8.62* (8.28, 8.97)	225	3.74* (3.43, 4.07)	172	2.34* (2.08, 2.62)	1344	5.87* (5.67, 6.08)

Abbreviations: CI, confidence interval; COPD, chronic obstructive pulmonary disease; HCC, hepatocellular carcinoma; NA, not applicable; SMR, standard mortality ratio.

* *p* < 0.05.

### 3.2 The burden of HCC deaths in the United States

**FIGURE 2:**
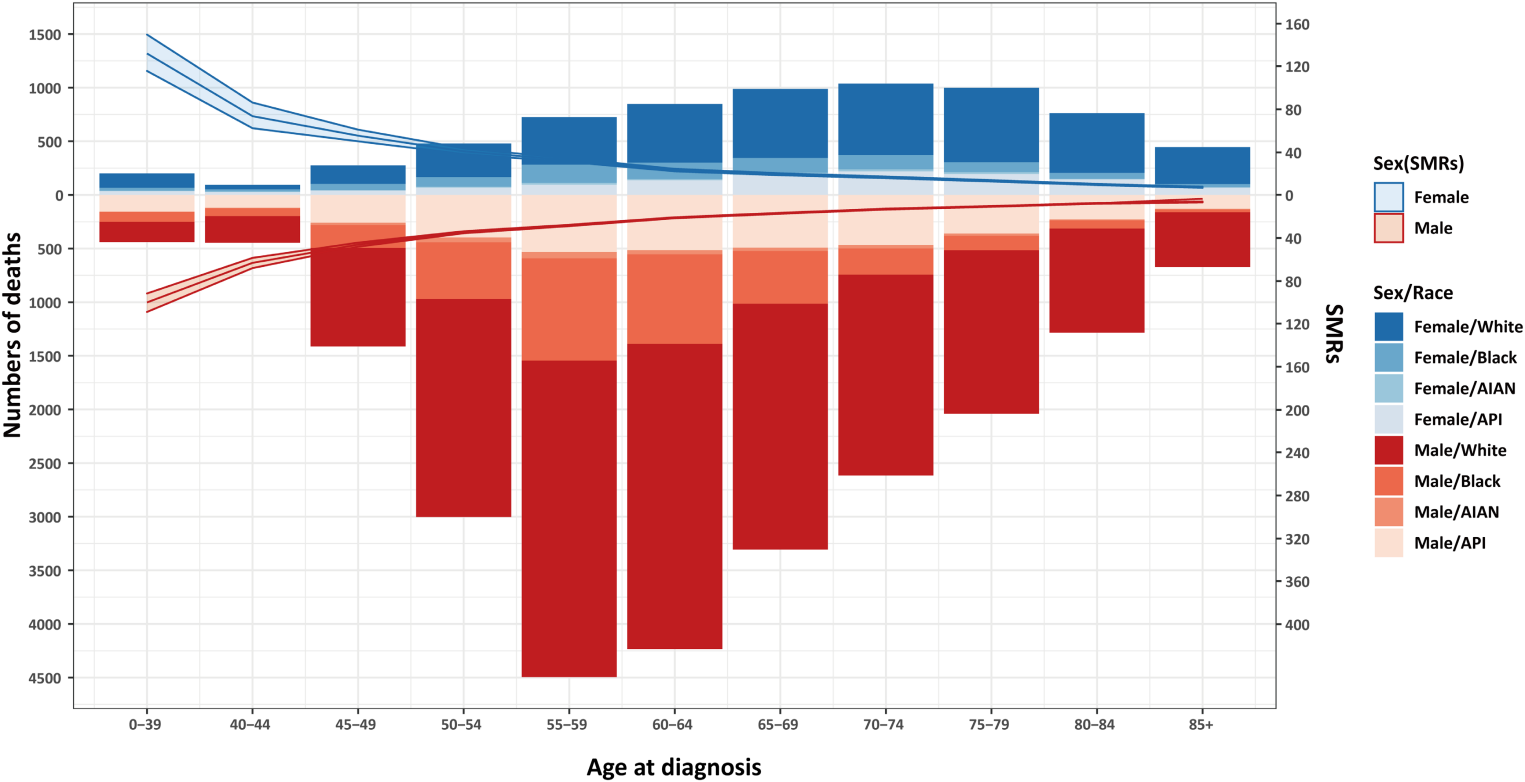
Age distribution of deaths among patients with hepatocellular carcinoma in United States. The bar was the number of all‐cause deaths in females and males, and the line with 95% confidence interval represents standard mortality ratios (SMRs). AIAN, American Indian or Alaska Native; API, Asian or Pacific Islander.

Deaths from all causes in patients with HCC occur disproportionately in men and women. The number of all‐cause deaths first increased and then decreased with age synchronously in both men and women, with the peak point appearing in men aged 55–59 years and women of 70–74 years, and more age‐specific deaths occurred in men compared to in women. Caucasian patients had the largest number of HCC‐related deaths, regardless of sex. Compared to the general US population, the relative risk of death due to any cause was significantly higher in male and female patients, while both experienced a significant decline in SMRs with advancing age (Figure 2).

### 3.3 Deaths per calendar year

**FIGURE 3:**
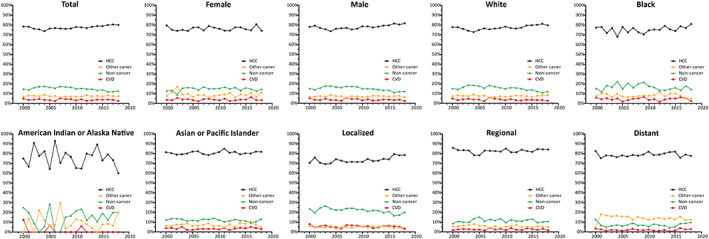
Plots of patient death versus attained calendar year (from 2000 to 2019), for HCC. Death was characterized as due to “HCC,” “Other cancers,” and “non‐cancer death.” CVD, cardiovascular disease; HCC, hepatocellular carcinoma.

Figure 3 shows the death rates in patients with HCC by calendar year from 2000 to 2018. Most deaths in the study population were due to HCC during each calendar year. Historical trends in HCC‐specific mortality indicate that the prognosis is still unfavorable, despite advancements in cancer treatment. The high mortality rate from HCC during the period 2000–2018 reflected the low risk of death due to causes other than HCC. The proportion of deaths due to non‐cancer causes in each calendar year is illustrated in Figure S1. Mortality rates as a result of the most non‐HCC causes remained relatively stable over the years, while there appeared to be a slight increase in the proportion of CVD‐related deaths in more recent years after cancer diagnosis.

### 3.4 CODs within 2 years after HCC diagnosis

A total of 25,153 deaths (81.7%) occurred within 2 years of HCC diagnosis. Of these, 19,994 (79.5%), 1938 (7.7%), and 3221 (12.8%) patients died of HCC, non‐HCC cancers, and non‐cancer causes, respectively. The most common non‐cancer CODs were other infectious and parasitic diseases, including HIV (905, 28.1%), followed by CVDs (771, 23.9%) and septicemia (119, 3.7%) (Table 2).

The overall risk of mortality within 2 years after HCC diagnosis was significantly higher compared with that of the general population, with a SMR of 34.67 (95% CI, 34.38–34.96), as was the risk of death due to other infectious and parasitic diseases, including HIV (SMR, 147.22; 95% CI, 141.82–152.78) and CVDs (SMR, 3.00; 95% CI, 2.85–3.15) (Table 2). Patients aged <50 years at diagnosis presented a risk elevation within 2 years of receiving an HCC diagnosis, as compared to those older than 50 years, with a significantly higher SMR of 202.87 in the younger groups. Patient demographics and tumor‐related trends of CODs among HCC survivors were similar (Tables [Link], [Link]). Non‐cancer causes constituted 268 of 2315 deaths (11.6%) among patients younger than 50 years of age within 2 years of HCC diagnosis. For patients aged 50–65 years, non‐cancer causes constituted 1546 of 11,105 deaths (13.9%) (Table S2). Among those older than 65 years, there were 1407 non‐cancer deaths, accounting for 12.6% of the total deaths in this subgroup (Table S3). Sex‐specific analysis showed that male patients had a significantly higher risk of all‐cause mortality than their female counterparts (SMR, 36.28 vs. 31.67) (Tables S4 and S5).

### 3.5 CODs from 2 to 5 years after HCC diagnosis

In total, 4075 patients with HCC died within 2–5 years of diagnosis, of whom 3001 (73.7%) died from HCC, 266 (6.5%) from other cancers, and 808 (19.8%) from non‐cancer causes. Other infectious and parasitic diseases, including HIV (205, 25.4%) and CVDs (195, 24.1%), were the two main non‐cancerous causes of mortality (Table 2). Overall, the SMR of all‐cause death at 2–5 years after an HCC diagnosis increased 11.26‐fold compared with the general US population (95% CI, 11.03–11.49).

### 3.6 CODs more than 5 years after HCC diagnosis

We observed 1568 deaths more than 5 years after cancer diagnosis, of which 829 (52.9%) were attributable to HCC, 85 (5.4%) to other cancers, and 654 (41.7%) to non‐cancer causes. CVDs (194, 29.7%) and other infectious and parasitic diseases, including HIV (104, 15.9%), were the major non‐cancer causes. Individuals with a latency of more than 5 years after an HCC diagnosis experienced strikingly an elevated risk of all‐cause death compared with the general US population (SMR, 4.47, 95% CI, 4.32–4.63) (Table 2).

### 3.7 Risks of non‐cancer death by patient subgroup

The mortality patterns of survivors of HCC as a function of patient age (Figure S2) and time after diagnosis (Figure S3) were examined in our study. A total of 4683 deaths were caused by non‐cancer diseases, of whom the plurality were caused by “CVDs,” “other infectious and parasitic diseases, including HIV,” and “other causes.” Our results indicated that the proportion of deaths attributable to different non‐cancer causes varied according to patient age group over the last two decades: those <30 years of age more frequently died from “other causes”; those aged between 30 and 65 years were more likely to die of “other infectious and parasitic diseases, including HIV” and “other causes”; and older (>65 years) patients were associated with a higher percentage of CVD‐related mortality (Figure S2). Subgroup analyses suggested that SMRs for some common non‐cancer causes followed a similar trend with patient age, with the highest SMRs being uniformly observed among younger individuals, except for the risk of death due to suicide and self‐inflicted injury (Figure S2). In contrast, subgroup analyses indicated that the proportions of most non‐cancer causes were more stable with increasing latency period, whereas a declining distribution was observed for other infectious and parasitic diseases, including HIV. CVDs and other causes were the predominant non‐cancer CODs across all follow‐up periods (Figure 3). SMRs plots according to the time after diagnosis showed that other infectious and parasitic diseases, including HIV, had the highest risk of death among all non‐cancer causes, whereas the magnitude of elevation in risk decreased dramatically with time after HCC diagnosis. Further analyses revealed that the relative risk of death due to nephritic/nephrotic diseases significantly increased beyond 5 years after cancer diagnosis (Figure S3).

## 4 DISCUSSION

In this study, we comprehensively analyzed the mortality patterns among individuals with HCC in a 20‐year cohort of patients in the United States. Our analyses revealed that the largest proportion of deaths occurred within 2 years of diagnosis. Although death from HCC was the most common cause of death, non‐cancer causes accounted for a substantial fraction of total deaths among older patients (≥50 years) compared with their younger counterparts (<50 years). The all‐cause mortality significantly exceeded that of the general US population. The highest non‐cancer SMRs were observed in other infectious and parasitic diseases, including HIV, followed by septicemia. Overall risks of death were generally attenuated for advanced‐stage HCC compared to early‐stage HCC and varied by selected patient subgroup, which offers a roadmap for targeted efforts to further mitigate the mortality burden after cancer diagnosis. In addition, the frequency of non‐cancer CODs changed over time, with infections, CVDs, and other non‐cancer CODs becoming more common after the diagnosis of HCC.

This longitudinal evaluation extensively examined for the first time the causes and risks of death in patients with HCC using data from a population‐based registry. Owing to stabilized HCC mortality and improved cancer management, a better understanding of CODs is of utmost importance because patients are more likely to survive long enough after an HCC diagnosis for the occurrence of non‐HCC‐related comorbidities.

Previous studies have delineated the CODs in patients with other cancer types.^19^, ^20^, ^21^, ^22^, ^23^ Our study is unparalleled as it characterizes CODs in individuals diagnosed with HCC using data from a large population‐based program as a function of latency after diagnosis and patient age group. Furthermore, the evaluation of SMRs in our study could help identify patients at a higher risk of death due to a specific cause and aid clinicians in clinical decision‐making.

Risk of non‐cancer mortality has been found to be significantly elevated in the group of cancer patients. Infections are a major non‐cancerous cause of morbidity and mortality. Increased infectious complications induced by complex immune dysfunction during cancer development and treatment have become a critical challenge for cancer survivors.^24^, ^25^ Our data revealed that patients with HCC were at a strikingly increased risk of death due to infectious diseases compared with the reference US population. Notably, other infectious and parasitic diseases, including HIV account for the highest number of deaths. Severe immunosuppression caused by HIV infection significantly increases the risk of infection‐related deaths among cancer survivors. Similarly, previous studies have also demonstrated that patients with Kaposi sarcoma, liver cancer, and lymphoma are associated with a more pronounced risk of infection‐related mortality.^17^, ^26^ Given the high mortality rates, close monitoring of these fatal infections and timely anti‐infective care are vital among individuals after a diagnosis of HCC.

The underlying risk factors for cancer development and treatment also put patients at risk of developing CVDs. In the United States, cancer and CVDs have become the leading causes of mortality among cancer survivors.^27^, ^28^, ^29^ In 2020, there were 912,681 and 602,372 deaths due to CVDs and malignant tumors, respectively. In view of this, CVDs has been one of the most studied non‐cancer causes of mortality across all time periods following cancer diagnosis.^30^, ^31^, ^32^ A large cohort study assessing mortality in the United States found that cancer survivors had an elevated risk of CVDs mortality compared with the general US population. Of note, the risk of death due to CVDs has surpassed cancer‐specific mortality risk in some cancer types, such as prostate cancer, thyroid cancer, and Hodgkin's lymphoma.^33^ An observational retrospective study by Carballo‐Folgoso et al.^34^ evaluated the frequency of cardiovascular events in 299 patients with advanced HCC and reported that sorafenib was associated with a significantly increased risk of CVDs. Consistent with previous studies, our analysis showed that CVDs were among the most pronounced causes of non‐cancer deaths with respect to continuous calendar year and follow‐up time after diagnosis. These findings underscore the importance of multidisciplinary care and early intervention by cardiologists among cancer survivors.

Previous studies demonstrated that lung cancer, especially squamous cell carcinoma, is closely related to COPD, as both share similar risk factors. However, the relationship between COPD and HCC has not been thoroughly investigated. We reported that HCC survivors had a higher SMR than the general population, and that patients aged 45–49 had the highest risk of death due to COPD. This phenomenon may be partially attributed to smoking tobacco. Our findings further emphasize the importance of targeting COPD prevention strategies in patients with HCC.

Our results indicate significant racial disparities and differences with respect to the risk of mortality at the population level. According to our study, AIAN patients were found to be associated with a significant higher SMR compared with other racial groups. AIANs have been reported to have the worst survival outcomes among all cancer patients in the United States, which could partly be attributed to limited access to optimal therapies and inequities in the delivery of cancer care.^35^ The worse survival results warrant a more intensive analysis to clarify and eliminate the barriers in this population.

The strengths of our analyses include the large sample size, population‐based settings, and long‐term follow‐up period. However, this study has some limitations. First, CODs, as confirmed by death certificates, may be miscoded, which could result in an over‐ or underestimation of the true number of cause‐specific deaths. Second, individual‐level data, including performance status and underlying diseases were not available in the SEER database. Third, the absence of treatment details, including chemotherapy regimens and radiation doses, which have been demonstrated to be closely associated with CVDs, may preclude exploring the effects of therapies on the relationship between cardiovascular events and cancer diagnosis. Additionally, cases with a more recent cancer diagnosis had a short follow‐up period, which limited the power to analyze temporal trends in CODs. Finally, our study only focuses on patients with a histological diagnosis of HCC as their first primary malignancy, and thus the number of patients identified in our study does not represent the total number of liver cancer cases in the United States. Nevertheless, the selection criteria we used in our study were necessary to ensure the accuracy and reliability of our results.

## 5 CONCLUSIONS

In summary, our analyses of the SEER‐18 database show that although patients with HCC are most likely to die of cancer, there is an increase in the number of non‐cancer deaths as the number of months since diagnosis increases. Other infectious and parasitic diseases, including HIV, are among the most common causes of non‐cancer‐related deaths. In addition, the increased risk of death attributable to CVDs continues to persist from the point of HCC diagnosis to survivorship, compared with the general US population. Further analyses also demonstrated profound racial differences and disparities with respect to mortality risk, as indicated by the higher death burden and more unfavorable prognosis among patients with AIAN. These encouraging findings highlight the need for greater holistic care and provide a good reference for how HCC survivors should be counseled regarding future health risks during cancer survivorship.

## AUTHOR CONTRIBUTIONS

**Zhen Yang:** Formal analysis (equal); software (lead); writing – original draft (lead); writing – review and editing (lead). **Kaiming Leng:** Data curation (equal); formal analysis (equal). **Guangjun Shi:** Conceptualization (lead).

## FUNDING INFORMATION

This study was supported by the Natural Science Foundation of Shandong Province (No. ZR2021MH172).

## CONFLICT OF INTEREST STATEMENT

The authors have no financial interests.

## ETHICS STATEMENT

Informed consent forms were waived as the data were derived from a publicly available database.

## Supporting information


Figure S1.

Figure S2.

Figure S3.

Table S1.

Table S2.

Table S3.

Table S4.

Table S5.

Table S6.

Table S7.

Table S8.

Table S9.

Table S10.

Table S11.

Table S12.

Table S13.

Table S14.

Table S15.

Legends

## Data Availability

The raw data in current study are available from the corresponding author.
