# Supplementary material for: Causes of death among patients with hepatocellular carcinoma in United States from 2000 to 2018
Source: Cancer Med. 2023 Apr 21;12(12):13076–85. doi: 10.1002/cam4.5986 (PMC10315789; doi:10.1002/cam4.5986)
Supplement: Supplementary file 4 — Table S1. [file CAM4-12-13076-s003.docx]

| **eTable 1. SMRs for each cause of death following HCC diagnosis in patients younger than 50 years.** | | | | | | | | | | | |
| --- | --- | --- | --- | --- | --- | --- | --- | --- | --- | --- | --- |
| **Cause of death** | **Deaths by time after diagnosis** | | | | | | | | | **Total deaths** | |
|  | **<2y** | |  | **2-5y** | |  | **>5y** | | |  |  |
|  | **Observed,**  **No.** | **SMR**  **(95% CI)** |  | **Observed,**  **No.** | **SMR**  **(95% CI)** |  | **Observed,**  **No.** | **SMR**  **(95% CI)** |  | **Observed,**  **No.** | **SMR**  **(95% CI)** |
| All | 2315 | 202.87*  (197.17, 208.69) |  | 369 | 39.69*  (36.90, 42.63) |  | 183 | 8.49*  (7.60, 9.47) |  | 2867 | 72.58*  (70.74, 74.46) |
| HCC | 1890 | NA |  | 292 | NA |  | 85 | NA |  | 2267 | NA |
| Other cancers | 157 | 61.31*  (54.28, 69.00) |  | 28 | 13.65*  (10.28, 17.77) |  | 8 | 2.91*  (1.93, 4.20) |  | 193 | 19.73*  (17.74, 21.88) |
| Non-cancer causes | 268 | 49.60*  (46.46, 52.89) |  | 49 | 12.52*  (10.77, 14.48) |  | 90 | 5.38*  (4.55, 6.32) |  | 407 | 20.69*  (19.57, 21.87) |
| Cardiovascular diseases | 32 | 9.62*  (7.27, 12.50) |  | 7 | 2.69*(1.43, 4.61) |  | 15 | 2.01*  (1.24, 3.07) |  | 54 | 4.27*  (3.43, 5.24) |
| Septicemia | 6 | 45.71*  (23.62, 79.84) |  | 0 | 4.47  (0.11, 24.92) |  | 3 | 9.85*  (3.20, 22.99) |  | 9 | 18.12*  (10.74, 26.83) |
| Pneumonia and Influenza | 1 | 11.22*  (2.31, 32.79) |  | 1 | 4.56  (0.12, 25.40) |  | 2 | 6.20*  (1.28, 18.12) |  | 4 | 7.21*  (2.90, 14.86) |
| COPD | 1 | 11.14*  (3.03, 28.51) |  | 1 | 2.74  (0.07, 15.26) |  | 2 | 1.65  (0.20, 5.96) |  | 4 | 3.62*  (1.45, 7.45) |
| Other Infectious and Parasitic Diseases including HIV | 114 | 323.91*  (289.49, 361.29) |  | 22 | 100.54*  (77.43, 128.39) |  | 25 | 34.67*  (23.71, 48.94) |  | 161 | 163.68*  (148.37, 180.15) |
| Diabetes Mellitus | 0 | 8.38*  (3.08, 18.24) |  | 0 | 1.62  (0.04, 9.03) |  | 5 | 7.52*  (3.75, 13.45) |  | 5 | 6.44*  (3.81, 10.17) |
| Nephritis, Nephrotic Syndrome and Nephrosis | 7 | 28.04*  (11.27, 57.77) |  | 0 | 9.59*  (1.16, 34.64) |  | 6 | 14.06*  (5.65, 28.98) |  | 13 | 16.74*  (9.57, 27.18) |
| Accidents and adverse effects of medications | 8 | 4.21*  (2.36, 6.95) |  | 3 | 3.21*  (1.39, 6.33) |  | 7 | 2.86*  (1.37, 5.26) |  | 18 | 3.46*  (2.38, 4.86) |
| Suicide and Self-Inflicted Injury | 4 | 2.69  (0.73, 6.89) |  | 1 | 0.94  (0.02, 5.25) |  | 0 | 1.47  (0.01, 2.51) |  | 5 | 1.24  (0.40, 2.90) |
| Other | 95 | 69.19*  (59.72, 79.73) |  | 14 | 13.61*  (9.18, 19.43) |  | 25 | 8.15*  (5.71, 11.29) |  | 134 | 27.40*  (24.15, 30.96) |
| **SMR, standard mortality ratio; HCC, hepatocellular carcinoma; COPD,chronic obstructive pulmonary disease; NA, not applicable; CI, confidence interval. * P < 0.05.** | | | | | | | | | | | |
