# Supplementary material for: Causes of death among patients with hepatocellular carcinoma in United States from 2000 to 2018
Source: Cancer Med. 2023 Apr 21;12(12):13076–85. doi: 10.1002/cam4.5986 (PMC10315789; doi:10.1002/cam4.5986)
Supplement: Supplementary file 5 — Table S2. [file CAM4-12-13076-s004.docx]

| **eTable 2. SMRs for each cause of death following HCC diagnosis in patients aged 50-65 years.** | | | | | | | | | | | |
| --- | --- | --- | --- | --- | --- | --- | --- | --- | --- | --- | --- |
| **Cause of death** | **Deaths by time after diagnosis** | | | | | | | | | **Total deaths** | |
|  | **<2y** | |  | **2-5y** | |  | **>5y** | | |  |  |
|  | **Observed,**  **No.** | **SMR**  **(95% CI)** |  | **Observed,**  **No.** | **SMR**  **(95% CI)** |  | **Observed,**  **No.** | **SMR**  **(95% CI)** |  | **Observed,**  **No.** | **SMR**  **(95% CI)** |
| All | 11105 | 62.31*  (61.56, 63.07) |  | 1902 | 16.65*  (16.18, 17.14) |  | 777 | 5.19*  (4.94, 5.45) |  | 13784 | 32.29*  (31.94, 32.65) |
| HCC | 8759 | NA |  | 1344 | NA |  | 399 | NA |  | 10502 | NA |
| Other cancers | 800 | 13.25*  (12.61, 13.92) |  | 140 | 4.47*  (4.02, 4.95) |  | 44 | 2.32*  (2.02, 2.66) |  | 984 | 7.40*  (7.10, 7.72) |
| Non-cancer causes | 1546 | 15.09*  (14.63, 15.56) |  | 418 | 5.62*  (5.28, 5.98) |  | 334 | 2.98*  (2.74, 3.23) |  | 2298 | 8.79*  (8.57, 9.02) |
| Cardiovascular diseases | 260 | 4.40*  (4.04, 4.79) |  | 79 | 2.09*  (1.79, 2.43) |  | 92 | 1.63*  (1.38, 1.92) |  | 431 | 2.93*  (2.74, 3.13) |
| Septicemia | 50 | 19.71*  (16.34, 23.57) |  | 15 | 9.17*  (6.47, 12.55) |  | 13 | 4.07*  (2.45, 6.36) |  | 78 | 11.87*  (10.19, 13.75) |
| Pneumonia and Influenza | 17 | 7.48*  (5.37, 10.14) |  | 8 | 4.42*  (2.57, 7.07) |  | 7 | 2.48*  (1.28, 4.33) |  | 32 | 4.94*  (3.85, 6.24) |
| COPD | 32 | 4.68*  (3.71, 5.83) |  | 6 | 1.16  (0.65, 1.91) |  | 13 | 1.17*  (1.15, 2.44) |  | 51 | 2.62*  (2.18, 3.12) |
| Other Infectious and Parasitic Diseases including HIV | 604 | 198.98*  (190.01, 208.26) |  | 141 | 71.57*  (64.42, 79.30) |  | 64 | 33.44*  (28.04, 39.58) |  | 809 | 127.48*  (122.38, 132.74) |
| Diabetes Mellitus | 41 | 4.70*  (3.73, 5.85) |  | 19 | 3.19*  (2.25, 4.40) |  | 18 | 2.36*  (1.59, 3.37) |  | 78 | 3.56*  (3.00, 4.18) |
| Nephritis, Nephrotic Syndrome and Nephrosis | 29 | 12.05*  (9.55, 14.99) |  | 9 | 5.43*  (3.51, 8.01) |  | 23 | 6.40*  (4.45, 8.89) |  | 61 | 8.37*  (7.04, 9.88) |
| Accidents and adverse effects of medications | 59 | 6.13*  (5.17, 7.23) |  | 21 | 4.00*  (2.99, 5.23) |  | 19 | 2.84*  (1.94, 4.01) |  | 99 | 4.76*  (4.16, 5.42) |
| Suicide and Self-Inflicted Injury | 14 | 2.49*  (1.58, 3.73) |  | 1 | 1.34  (0.54, 2.75) |  | 4 | 1.43  (0.53, 3.12) |  | 19 | 1.93*  (1.35, 2.67) |
| Other | 440 | 22.99*  (21.62, 24.43) |  | 119 | 8.20*  (7.22, 9.27) |  | 81 | 3.45*  (2.88, 4.11) |  | 640 | 12.61*  (11.97, 13.28) |
| **SMR, standard mortality ratio; HCC, hepatocellular carcinoma; COPD,chronic obstructive pulmonary disease; NA, not applicable; CI, confidence interval. * P < 0.05.** | | | | | | | | | | | |
