# Supplementary material for: Causes of death among patients with hepatocellular carcinoma in United States from 2000 to 2018
Source: Cancer Med. 2023 Apr 21;12(12):13076–85. doi: 10.1002/cam4.5986 (PMC10315789; doi:10.1002/cam4.5986)
Supplement: Supplementary file 6 — Table S3. [file CAM4-12-13076-s011.docx]

| **eTable 3. SMRs for each cause of death following HCC diagnosis in patients older than 65 years.** | | | | | | | | | | | |
| --- | --- | --- | --- | --- | --- | --- | --- | --- | --- | --- | --- |
| **Cause of death** | **Deaths by time after diagnosis** | | | | | | | | | **Total deaths** | |
|  | **<2y** | |  | **2-5y** | |  | **>5y** | | |  |  |
|  | **Observed,**  **No.** | **SMR**  **(95% CI)** |  | **Observed,**  **No.** | **SMR**  **(95% CI)** |  | **Observed,**  **No.** | **SMR**  **(95% CI)** |  | **Observed,**  **No.** | **SMR**  **(95% CI)** |
| All | 11173 | 21.35*  (21.08, 21.62) |  | 1804 | 7.50*  (7.27, 7.73) |  | 608 | 3.43*  (3.24, 3.63) |  | 14145 | 14.65*  (14.48, 14.81) |
| HCC | 9345 | NA |  | 1365 | NA |  | 345 | NA |  | 11055 | NA |
| Other cancers | 981 | 6.94*  (6.64, 7.26) |  | 98 | 2.35*  (2.09, 2.63) |  | 33 | 1.74*  (1.45, 2.07) |  | 1112 | 4.94*  (4.74, 5.14) |
| Non-cancer causes | 1407 | 3.93*  (3.80, 4.06) |  | 341 | 1.98*  (1.85, 2.13) |  | 230 | 1.51*  (1.37, 1.67) |  | 1978 | 2.99*  (2.90, 3.08) |
| Cardiovascular diseases | 479 | 2.47*  (2.32, 2.62) |  | 109 | 1.28*  (1.12, 1.45) |  | 87 | 1.26*  (1.07, 1.47) |  | 675 | 1.95*  (1.85, 2.06) |
| Septicemia | 63 | 7.06*  (5.87, 8.44) |  | 11 | 3.02*  (1.93, 4.49) |  | 6 | 1.98  (0.95, 3.63) |  | 80 | 5.15*  (4.37, 6.03) |
| Pneumonia and Influenza | 35 | 2.36*  (1.84, 2.99) |  | 14 | 1.80*  (1.16, 2.66) |  | 7 | 1.30  (0.69, 2.22) |  | 56 | 2.01*  (1.65, 2.43) |
| COPD | 67 | 1.79*  (1.50, 2.12) |  | 19 | 1.11  (0.79, 1.53) |  | 17 | 1.37  (0.92, 1.95) |  | 103 | 1.54*  (1.34, 1.77) |
| Other Infectious and Parasitic Diseases including HIV | 187 | 72.14*  (66.62, 77.99) |  | 42 | 31.99*  (26.69, 38.04) |  | 15 | 15.95*  (11.35, 21.81) |  | 244 | 52.52*  (48.94, 56.30) |
| Diabetes Mellitus | 69 | 4.00*  (3.41, 4.68) |  | 22 | 1.79*  (1.23, 2.51) |  | 9 | 1.12  (0.60, 1.91) |  | 100 | 2.94*  (2.55, 3.37) |
| Nephritis, Nephrotic Syndrome and Nephrosis | 53 | 4.31*  (3.53, 5.21) |  | 10 | 2.04*  (1.31, 3.04) |  | 13 | 2.42*  (1.46, 3.78) |  | 76 | 3.38*  (2.86, 3.96) |
| Accidents and adverse effects of medications | 37 | 3.28*  (2.66, 4.01) |  | 17 | 2.88*  (2.05, 3.93) |  | 9 | 1.54  (0.84, 2.58) |  | 63 | 2.87*  (2.42, 3.37) |
| Suicide and Self-Inflicted Injury | 5 | 2.40*  (1.35, 3.97) |  | 5 | 2.61*  (1.05, 5.38) |  | 1 | 2.12  (0.44, 6.21) |  | 11 | 2.42*  (1.57, 3.57) |
| Other | 412 | 5.47*  (5.12, 5.83) |  | 92 | 2.62*  (2.28, 3.00) |  | 66 | 1.62*  (1.31, 1.98) |  | 570 | 3.98*  (3.76, 4.21) |
| **SMR, standard mortality ratio; HCC, hepatocellular carcinoma; COPD,chronic obstructive pulmonary disease; NA, not applicable; CI, confidence interval. * P < 0.05.** | | | | | | | | | | | |
