# Supplementary material for: Causes of death among patients with hepatocellular carcinoma in United States from 2000 to 2018
Source: Cancer Med. 2023 Apr 21;12(12):13076–85. doi: 10.1002/cam4.5986 (PMC10315789; doi:10.1002/cam4.5986)
Supplement: Supplementary file 7 — Table S4. [file CAM4-12-13076-s002.docx]

| **eTable 4. SMRs for each cause of death following HCC diagnosis in female patients.** | | | | | | | | | | | |
| --- | --- | --- | --- | --- | --- | --- | --- | --- | --- | --- | --- |
| **Cause of death** | **Deaths by time after diagnosis** | | | | | | | | | **Total deaths** | |
|  | **<2y** | |  | **2-5y** | |  | **>5y** | | |  |  |
|  | **Observed,**  **No.** | **SMR**  **(95% CI)** |  | **Observed,**  **No.** | **SMR**  **(95% CI)** |  | **Observed,**  **No.** | **SMR**  **(95% CI)** |  | **Observed,**  **No.** | **SMR**  **(95% CI)** |
| All | 5549 | 31.67*  (31.11, 33.23) |  | 924 | 10.99*  (10.53, 11.46) |  | 377 | 4.56*  (4.24, 4.89) |  | 6850 | 20.13*  (19.81, 20.45) |
| HCC | 4323 | NA |  | 697 | NA |  | 201 | NA |  | 5221 | NA |
| Other cancers | 506 | 10.63*  (9.97, 11.33) |  | 65 | 3.50*  (2.98, 4.07) |  | 20 | 2.17*  (1.74, 2.69) |  | 591 | 6.82*  (6.44, 7.21) |
| Non-cancer causes | 720 | 6.38*  (6.10, 6.68) |  | 162 | 2.96*  (2.69, 3.25) |  | 156 | 2.30*  (2.04, 2.58) |  | 1038 | 4.57*  (4.40, 4.75) |
| Cardiovascular diseases | 181 | 2.95*  (2.67, 3.27) |  | 39 | 1.70*  (1.39, 2.05) |  | 48 | 1.55*  (1.24, 1.93) |  | 268 | 2.32*  (2.14, 2.52) |
| Septicemia | 24 | 9.97*  (7.64, 12.78) |  | 10 | 7.13*  (4.52, 10.70) |  | 7 | 4.42*  (2.29, 7.73) |  | 41 | 7.98*  (6.47, 9.73) |
| Pneumonia and Influenza | 11 | 3.05*  (2.04, 4.38) |  | 2 | 1.45  (0.58, 2.99) |  | 4 | 2.18  (1.00, 4.14) |  | 17 | 2.44*  (1.78, 3.26) |
| COPD | 24 | 1.91*  (1.39, 2.55) |  | 4 | 0.83  (0.40, 1.52) |  | 9 | 2.27*  (1.45, 3.38) |  | 37 | 1.71*  (1.35, 2.13) |
| Other Infectious and Parasitic Diseases including HIV | 187 | 168.24*  (154.71, 182.64) |  | 36 | 60.88*  (50.09, 73.32) |  | 17 | 30.16*  (22.00, 40.36) |  | 240 | 108.31*  (100.57, 116.48) |
| Diabetes Mellitus | 26 | 4.56*  (3.49, 5.86) |  | 8 | 2.27*  (1.30, 3.68) |  | 9 | 1.69  (0.81, 3.11) |  | 43 | 3.30*  (2.65, 4.07) |
| Nephritis, Nephrotic Syndrome and Nephrosis | 26 | 6.86*  (5.17, 8.93) |  | 6 | 2.62*  (1.31, 4.69) |  | 13 | 4.78*  (2.79, 7.66) |  | 45 | 5.26*  (4.19, 6.53) |
| Accidents and adverse effects of medications | 22 | 5.46*  (4.07, 7.15) |  | 6 | 2.73*  (1.49, 4.57) |  | 4 | 2.28*  (1.09, 4.19) |  | 32 | 3.99*  (3.14, 4.99) |
| Suicide and Self-Inflicted Injury | 1 | 1.72  (0.21, 6.21) |  | 1 | 1.49  (0.04, 8.29) |  | 0 | 0.54  (0.00, 6.78) |  | 2 | 1.26  (0.26, 3.69) |
| Other | 218 | 8.57*  (7.85, 9.33) |  | 50 | 3.87*  (3.22, 4.60) |  | 45 | 2.30*  (1.78, 2.92) |  | 313 | 5.87*  (5.45, 6.31) |
| **SMR, standard mortality ratio; HCC, hepatocellular carcinoma; COPD,chronic obstructive pulmonary disease; NA, not applicable; CI, confidence interval. * P < 0.05.** | | | | | | | | | | | |
