# Supplementary material for: Causes of death among patients with hepatocellular carcinoma in United States from 2000 to 2018
Source: Cancer Med. 2023 Apr 21;12(12):13076–85. doi: 10.1002/cam4.5986 (PMC10315789; doi:10.1002/cam4.5986)
Supplement: Supplementary file 8 — Table S5. [file CAM4-12-13076-s012.docx]

| **eTable 5. SMRs for each cause of death following HCC diagnosis in male patients.** | | | | | | | | | | | |
| --- | --- | --- | --- | --- | --- | --- | --- | --- | --- | --- | --- |
| **Cause of death** | **Deaths by time after diagnosis** | | | | | | | | | **Total deaths** | |
|  | **<2y** | |  | **2-5y** | |  | **>5y** | | |  |  |
|  | **Observed,**  **No.** | **SMR**  **(95% CI)** |  | **Observed,**  **No.** | **SMR**  **(95% CI)** |  | **Observed,**  **No.** | **SMR**  **(95% CI)** |  | **Observed,**  **No.** | **SMR**  **(95% CI)** |
| All | 19604 | 36.28*  (35.95, 36.62) |  | 3151 | 11.66*  (11.39, 11.93) |  | 1191 | 4.56*  (4.38, 4.75) |  | 23946 | 22.71*  (22.52, 22.90) |
| HCC | 15671 | NA |  | 2304 | NA |  | 628 | NA |  | 18603 | NA |
| Other cancers | 1432 | 9.09*  (8.76, 9.43) |  | 201 | 3.36*  (3.08, 3.65) |  | 65 | 2.09*  (1.85, 2.35) |  | 1698 | 6.02*  (5.82, 6.21) |
| Non-cancer causes | 2501 | 7.34*  (7.16, 7.52) |  | 646 | 3.35*  (3.19, 3.53) |  | 498 | 2.28*  (2.13, 2.44) |  | 3645 | 5.16*  (5.05, 5.27) |
| Cardiovascular diseases | 590 | 3.01*  (2.85, 3.19) |  | 156 | 1.51*  (1.35, 1.68) |  | 146 | 1.41*  (1.24, 1.60) |  | 892 | 2.27*  (2.16, 2.38) |
| Septicemia | 95 | 11.03*  (9.53, 12.71) |  | 16 | 4.39*  (3.14, 5.98) |  | 15 | 2.92*  (1.83, 4.43) |  | 126 | 7.46*  (6.57, 8.44) |
| Pneumonia and Influenza | 42 | 3.30*  (2.63, 4.09) |  | 21 | 2.74*  (1.92, 3.79) |  | 12 | 1.69*  (1.02, 2.64) |  | 75 | 2.79*  (2.35, 3.29) |
| COPD | 76 | 2.52*  (2.15, 2.92) |  | 22 | 1.24  (0.90, 1.67) |  | 23 | 1.26  (0.89, 1.73) |  | 121 | 1.90*  (1.67, 2.14) |
| Other Infectious and Parasitic Diseases including HIV | 718 | 142.67*  (136.82, 148.72) |  | 169 | 56.35*  (51.24, 61.83) |  | 87 | 27.23*  (23.19, 31.78) |  | 974 | 96.34*  (92.83, 99.95) |
| Diabetes Mellitus | 84 | 4.17*  (3.59, 4.82) |  | 33 | 2.33*  (1.75, 3.03) |  | 23 | 2.21*  (1.61, 2.97) |  | 140 | 3.23*  (2.86, 3.63) |
| Nephritis, Nephrotic Syndrome and Nephrosis | 63 | 5.87*  (4.93, 6.93) |  | 13 | 3.23*  (2.31, 4.40) |  | 29 | 4.28*  (3.11, 5.75) |  | 105 | 4.81*  (4.20, 5.49) |
| Accidents and adverse effects of medications | 82 | 4.34*  (3.76, 4.98) |  | 35 | 3.56*  (2.85, 4.39) |  | 31 | 2.36*  (1.73, 3.15) |  | 148 | 3.70*  (3.31, 4.12) |
| Suicide and Self-Inflicted Injury | 22 | 2.53*  (1.81, 3.44) |  | 6 | 1.69  (0.92, 2.83) |  | 5 | 1.38  (0.63, 2.62) |  | 33 | 2.06*  (1.58, 2.63) |
| Other | 729 | 10.53*  (10.03, 11.06) |  | 175 | 4.52*  (4.08, 5.01) |  | 127 | 2.70*  (2.33, 3.11) |  | 1031 | 7.12*  (6.82, 7.42) |
| **SMR, standard mortality ratio; HCC, hepatocellular carcinoma; COPD,chronic obstructive pulmonary disease; NA, not applicable; CI, confidence interval. * P < 0.05.** | | | | | | | | | | | |
