# Supplementary material for: Causes of death among patients with hepatocellular carcinoma in United States from 2000 to 2018
Source: Cancer Med. 2023 Apr 21;12(12):13076–85. doi: 10.1002/cam4.5986 (PMC10315789; doi:10.1002/cam4.5986)
Supplement: Supplementary file 9 — Table S6. [file CAM4-12-13076-s009.docx]

| **eTable 6. SMRs for each cause of death following HCC diagnosis in White patients.** | | | | | | | | | | | |
| --- | --- | --- | --- | --- | --- | --- | --- | --- | --- | --- | --- |
| **Cause of death** | **Deaths by time after diagnosis** | | | | | | | | | **Total deaths** | |
|  | **<2y** | |  | **2-5y** | |  | **>5y** | | |  |  |
|  | **Observed,**  **No.** | **SMR**  **(95% CI)** |  | **Observed,**  **No.** | **SMR**  **(95% CI)** |  | **Observed,**  **No.** | **SMR**  **(95% CI)** |  | **Observed,**  **No.** | **SMR**  **(95% CI)** |
| All | 17158 | 34.19*  (33.85, 34.53) |  | 2721 | 11.40*  (11.12, 11.68) |  | 1037 | 4.40*  (4.21, 4.59) |  | 20916 | 21.81*  (21.61, 22.00) |
| HCC | 13650 | NA |  | 1976 | NA |  | 501 | NA |  | 16127 | NA |
| Other cancers | 1316 | 8.94*  (8.60, 9.30) |  | 171 | 3.20*  (2.91, 3.50) |  | 51 | 2.10*  (1.85, 2.38) |  | 1538 | 5.95*  (5.75, 6.15) |
| Non-cancer causes | 2192 | 7.01*  (6.83, 7.19) |  | 574 | 3.35*  (3.18, 3.54) |  | 485 | 2.47*  (2.31, 2.65) |  | 3251 | 5.08*  (4.97, 5.20) |
| Cardiovascular diseases | 504 | 2.86*  (2.69, 3.03) |  | 141 | 1.55*  (1.37, 1.74) |  | 142 | 1.60*  (1.40, 1.82) |  | 787 | 2.26*  (2.15, 2.37) |
| Septicemia | 85 | 11.03*  (9.45, 12.80) |  | 17 | 6.01*  (4.43, 7.96) |  | 17 | 3.86*  (2.52, 5.65) |  | 119 | 8.13*  (7.15, 9.21) |
| Pneumonia and Influenza | 31 | 3.19*  (2.51, 4.01) |  | 18 | 2.81*  (1.91, 3.99) |  | 10 | 2.03*  (1.20, 3.20) |  | 59 | 2.86*  (2.37, 3.41) |
| COPD | 74 | 2.28*  (1.95, 2.65) |  | 20 | 1.05  (0.75, 1.44) |  | 22 | 1.449*  (1.09, 1.98) |  | 116 | 1.78*  (1.57, 2.02) |
| Other Infectious and Parasitic Diseases including HIV | 614 | 176.00*  (168.37, 183.90) |  | 142 | 67.33*  (60.83, 74.34) |  | 72 | 30.28*  (25.44, 35.78) |  | 828 | 116.30*  (11.81, 120.93) |
| Diabetes Mellitus | 74 | 4.50*  (3.82, 5.25) |  | 26 | 2.33*  (1.68, 3.15) |  | 24 | 2.80*  (2.02, 3.78) |  | 124 | 3.55*  (3.12, 4.03) |
| Nephritis, Nephrotic Syndrome and Nephrosis | 56 | 6.00*  (4.97, 7.18) |  | 12 | 3.65*  (2.56, 5.06) |  | 33 | 5.72*  (4.20, 7.61) |  | 101 | 5.33*  (4.62, 6.12) |
| Accidents and adverse effects of medications | 71 | 4.43*  (3.81, 5.13) |  | 32 | 3.70*  (2.92, 4.63) |  | 28 | 2.82*  (2.07, 3.75) |  | 131 | 3.89*  (3.47, 4.36) |
| Suicide and Self-Inflicted Injury | 18 | 2.48*  (1.74, 3.43) |  | 6 | 1.72  (0.91, 2.94) |  | 3 | 1.19  (0.48, 2.45) |  | 27 | 2.00*  (1.51, 2.60) |
| Other | 665 | 9.83*  (9.35, 10.33) |  | 160 | 4.42*  (3.97, 4.90) |  | 134 | 2.74*  (2.36, 3.16) |  | 959 | 6.82*  (6.53, 7.12) |
| **SMR, standard mortality ratio; HCC, hepatocellular carcinoma; COPD,chronic obstructive pulmonary disease; NA, not applicable; CI, confidence interval. * P < 0.05.** | | | | | | | | | | | |
