# Supplementary material for: Causes of death among patients with hepatocellular carcinoma in United States from 2000 to 2018
Source: Cancer Med. 2023 Apr 21;12(12):13076–85. doi: 10.1002/cam4.5986 (PMC10315789; doi:10.1002/cam4.5986)
Supplement: Supplementary file 10 — Table S7. [file CAM4-12-13076-s007.docx]

| **eTable 7. SMRs for each cause of death following HCC diagnosis in black patients.** | | | | | | | | | | | |
| --- | --- | --- | --- | --- | --- | --- | --- | --- | --- | --- | --- |
| **Cause of death** | **Deaths by time after diagnosis** | | | | | | | | | **Total deaths** | |
|  | **<2y** | |  | **2-5y** | |  | **>5y** | | |  |  |
|  | **Observed,**  **No.** | **SMR**  **(95% CI)** |  | **Observed,**  **No.** | **SMR**  **(95% CI)** |  | **Observed,**  **No.** | **SMR**  **(95% CI)** |  | **Observed,**  **No.** | **SMR**  **(95% CI)** |
| All | 3933 | 37.91*  (37.10, 38.73) |  | 525 | 11.46*  (10.82, 12.12) |  | 179 | 5.26*  (4.72, 5.85) |  | 4637 | 25.34*  (24.84, 25.84) |
| HCC | 3008 | NA |  | 376 | NA |  | 100 | NA |  | 3484 | NA |
| Other cancers | 324 | 11.21*  (10.35, 12.13) |  | 45 | 4.09*  (3.36, 4.93) |  | 8 | 1.35  (0.85, 2.05) |  | 377 | 7.65*  (7.11, 8.22) |
| Non-cancer causes | 601 | 8.61*  (8.16, 9.08) |  | 104 | 3.43*  (3.02, 3.88) |  | 71 | 2.55*  (2.11, 3.06) |  | 776 | 6.20*  (5.91, 6.50) |
| Cardiovascular diseases | 156 | 3.71*  (3.29, 4.16) |  | 25 | 1.81*  (1.40, 2.30) |  | 21 | 1.34  (0.91, 1.92) |  | 202 | 2.81*  (2.53, 3.11) |
| Septicemia | 21 | 11.45*  (8.58, 14.98) |  | 4 | 2.69  (0.99, 5.84) |  | 1 | 2.16  (0.45, 6.31) |  | 26 | 7.51*  (5.76, 9.63) |
| Pneumonia and Influenza | 9 | 2.39*  (1.09, 4.53) |  | 2 | 3.34*  (1.23, 7.28) |  | 1 | 0.90  (0.02, 5.01) |  | 12 | 2.40*  (1.37, 3.89) |
| COPD | 15 | 2.73*  (1.69, 4.17) |  | 1 | 1.55  (0.57, 3.38) |  | 5 | 2.42  (0.89, 5.26) |  | 21 | 2.35*  (1.62, 3.30) |
| Other Infectious and Parasitic Diseases including HIV | 169 | 89.31*  (81.20, 98.01) |  | 35 | 36.78*  (29.16, 45.77) |  | 15 | 26.45*  (18.09, 37.34) |  | 219 | 66.57*  (61.15, 72.33) |
| Diabetes Mellitus | 18 | 3.59*  (2.54, 4.93) |  | 5 | 1.95  (0.93, 3.58) |  | 4 | 1.57  (0.51, 3.65) |  | 27 | 2.80*  (2.10, 3.67) |
| Nephritis, Nephrotic Syndrome and Nephrosis | 20 | 6.43*  (4.63, 8.69) |  | 0 | 0.94  (0.19, 2.75) |  | 4 | 2.99*  (1.10, 6.52) |  | 24 | 4.35*  (3.24, 5.72) |
| Accidents and adverse effects of medications | 18 | 5.79*  (4.19, 7.80) |  | 4 | 2.91*  (1.39, 5.35) |  | 2 | 1.43  (0.30, 4.18) |  | 24 | 4.32*  (3.26, 5.61) |
| Suicide and Self-Inflicted Injury | 3 | 5.38*  (1.47, 13.77) |  | 1 | 6.06  (0.73, 21.90) |  | 2 | 10.39*  (1.26, 37.54) |  | 6 | 6.32*  (2.73, 12.45) |
| Other | 172 | 13.80*  (12.41, 15.31) |  | 27 | 4.68*  (3.57, 6.02) |  | 16 | 2.91*  (1.87, 4.34) |  | 215 | 9.41*  (8.56, 10.33) |
| **SMR, standard mortality ratio; HCC, hepatocellular carcinoma; COPD,chronic obstructive pulmonary disease; NA, not applicable; CI, confidence interval. * P < 0.05.** | | | | | | | | | | | |
