# Supplementary material for: Causes of death among patients with hepatocellular carcinoma in United States from 2000 to 2018
Source: Cancer Med. 2023 Apr 21;12(12):13076–85. doi: 10.1002/cam4.5986 (PMC10315789; doi:10.1002/cam4.5986)
Supplement: Supplementary file 11 — Table S8. [file CAM4-12-13076-s005.docx]

| **eTable 8. SMRs for each cause of death following HCC diagnosis in Indian American/Alaska Native patients.** | | | | | | | | | | | |
| --- | --- | --- | --- | --- | --- | --- | --- | --- | --- | --- | --- |
| **Cause of death** | **Deaths by time after diagnosis** | | | | | | | | | **Total deaths** | |
|  | **<2y** | |  | **2-5y** | |  | **>5y** | | |  |  |
|  | **Observed,**  **No.** | **SMR**  **(95% CI)** |  | **Observed,**  **No.** | **SMR**  **(95% CI)** |  | **Observed,**  **No.** | **SMR**  **(95% CI)** |  | **Observed,**  **No.** | **SMR**  **(95% CI)** |
| All | 305 | 74.68*  (69.34, 80.33) |  | 55 | 29.67*  (24.84, 35.16) |  | 18 | 10.18*  (7.09, 14.16) |  | 378 | 50.62*  (47.35, 54.05) |
| HCC | 237 | NA |  | 40 | NA |  | 9 | NA |  | 286 | NA |
| Other cancers | 27 | 18.42*  (13.49, 24.57) |  | 1 | 6.85*  (2.96, 13.50) |  | 0 | 2.24  (0.27, 8.10) |  | 28 | 12.29*  (9.28, 15.96) |
| Non-cancer causes | 41 | 15.22*  (12.40, 18.50) |  | 14 | 10.14*  (6.89, 14.39) |  | 9 | 5.98*  (3.27, 10.03) |  | 64 | 12.13*  (10.25, 14.27) |
| Cardiovascular diseases | 5 | 3.19*  (1.53, 5.88) |  | 1 | 1.41  (0.17, 5.09) |  | 2 | 3.68*  (1.00, 9.41) |  | 8 | 2.84*  (1.62, 4.61) |
| Septicemia | 1 | 15.52*  (1.88, 56.07) |  | 1 | 50.34*  (10.38, 147.11) |  | 0 | / |  | 2 | 21.38*  (6.94, 49.90) |
| Pneumonia and Influenza | 0 | / |  | 0 | / |  | 0 | / |  | 0 | / |
| COPD | 2 | 17.40  (6.39, 37.88) |  | 0 | / |  | 2 | 23.63*  (4.87, 69.06) |  | 4 | 14.29*  (6.53, 27.13) |
| Other Infectious and Parasitic Diseases including HIV | 10 | 257.20*  (178.12, 359.42) |  | 2 | 145.66*  (66.61, 276.52) |  | 1 | 43.59*  (5.28, 157.45) |  | 13 | 187.61*  (136.84, 251.03) |
| Diabetes Mellitus | 2 | 10.25*  (3.33, 23.93) |  | 1 | 4.37  (0.11, 24.36) |  | 0 | / |  | 3 | 6.72*  (2.47, 14.62) |
| Nephritis, Nephrotic Syndrome and Nephrosis | 0 | / |  | 0 | / |  | 0 | / |  | 0 | / |
| Accidents and adverse effects of medications | 3 | 18.90*  (7.60, 38.94) |  | 0 | / |  | 1 | 7.36  (0.19, 41.02) |  | 4 | 16.16*  (8.07, 28.91) |
| Suicide and Self-Inflicted Injury | 0 | / |  | 0 | / |  | 0 | / |  | 0 | / |
| Other | 18 | 27.96*  (19.25, 39.27) |  | 9 | 19.91*  (9.94, 35.63) |  | 3 | 9.52*  (2.59, 24.38) |  | 30 | 22.30*  (16.44, 29.57) |
| **SMR, standard mortality ratio; HCC, hepatocellular carcinoma; COPD,chronic obstructive pulmonary disease; NA, not applicable; CI, confidence interval. * P < 0.05.** | | | | | | | | | | | |
