# Supplementary material for: Causes of death among patients with hepatocellular carcinoma in United States from 2000 to 2018
Source: Cancer Med. 2023 Apr 21;12(12):13076–85. doi: 10.1002/cam4.5986 (PMC10315789; doi:10.1002/cam4.5986)
Supplement: Supplementary file 12 — Table S9. [file CAM4-12-13076-s010.docx]

| **eTable 9. SMRs for each cause of death following HCC diagnosis in Asian or Pacific Islander patients..** | | | | | | | | | | | |
| --- | --- | --- | --- | --- | --- | --- | --- | --- | --- | --- | --- |
| **Cause of death** | **Deaths by time after diagnosis** | | | | | | | | | **Total deaths** | |
|  | **<2y** | |  | **2-5y** | |  | **>5y** | | |  |  |
|  | **Observed,**  **No.** | **SMR**  **(95% CI)** |  | **Observed,**  **No.** | **SMR**  **(95% CI)** |  | **Observed,**  **No.** | **SMR**  **(95% CI)** |  | **Observed,**  **No.** | **SMR**  **(95% CI)** |
| All | 3757 | 36.79*  (36.02, 37.57) |  | 774 | 11.86*  (11.32, 12.42) |  | 334 | 4.77*  (4.44, 5.13) |  | 4865 | 20.57*  (20.20, 20.96) |
| HCC | 3099 | NA |  | 609 | NA |  | 219 | NA |  | 3927 | NA |
| Other cancers | 271 | 9.81*  (9.00, 10.67) |  | 49 | 3.55*  (2.96, 4.21) |  | 26 | 2.47*  (1.98, 3.04) |  | 346 | 5.95*  (5.53, 6.39) |
| Non-cancer causes | 387 | 5.82*  (5.46, 6.19) |  | 116 | 2.59*  (2.29, 2.90) |  | 89 | 1.55*  (1.33, 1.80) |  | 592 | 3.68*  (3.49, 3.87) |
| Cardiovascular diseases | 106 | 2.96*  (2.60, 3.36) |  | 28 | 1.39*  (1.09, 1.75) |  | 29 | 1.01  (0.75, 1.32) |  | 163 | 1.97*  (1.78, 2.18) |
| Septicemia | 12 | 8.09*  (5.23, 11.94) |  | 4 | 2.93*  (1.08, 6.38) |  | 4 | 2.43*  (0.79, 5.66) |  | 20 | 5.00*  (3.50, 6.92) |
| Pneumonia and Influenza | 13 | 3.86*  (2.61, 5.52) |  | 3 | 1.20  (0.44, 2.60) |  | 5 | 1.71  (0.78, 3.24) |  | 21 | 2.49*  (1.82, 3.34) |
| COPD | 9 | 2.15*  (1.33, 3.29) |  | 5 | 1.41  (0.64, 2.67) |  | 3 | 0.92  (0.34, 2.00) |  | 17 | 1.59*  (1.11, 2.20) |
| Other Infectious and Parasitic Diseases including HIV | 112 | 126.96*  (113.59, 141.47) |  | 26 | 45.27*  (35.67, 56.67) |  | 16 | 21.36*  (14.80, 29.85) |  | 154 | 74.83*  (67.98, 82.17) |
| Diabetes Mellitus | 16 | 3.90*  (2.82, 5.25) |  | 9 | 2.45*  (1.45, 3.88) |  | 4 | 0.94  (0.38, 1.94) |  | 29 | 2.64*  (2.05, 3.34) |
| Nephritis, Nephrotic Syndrome and Nephrosis | 13 | 6.01*  (4.09, 8.53) |  | 7 | 3.50*  (1.81, 6.11) |  | 5 | 2.27  (0.98, 4.46) |  | 25 | 4.21*  (3.13, 5.53) |
| Accidents and adverse effects of medications | 12 | 3.08*  (1.95, 4.62) |  | 5 | 2.04  (0.98, 3.75) |  | 4 | 1.01  (0.33, 2.36) |  | 21 | 2.19*  (1.55, 3.01) |
| Suicide and Self-Inflicted Injury | 2 | 1.24  (0.15, 4.49) |  | 0 | / |  | 0 | / |  | 2 | 0.56  (0.07, 2.01) |
| Other | 92 | 6.73*  (5.83, 7.72) |  | 29 | 3.38*  (2.63, 4.28) |  | 19 | 1.82*  (1.30, 2.48) |  | 140 | 4.30*  (3.84, 4.81) |
| **SMR, standard mortality ratio; HCC, hepatocellular carcinoma; COPD,chronic obstructive pulmonary disease; NA, not applicable; CI, confidence interval. * P < 0.05.** | | | | | | | | | | | |
