# Supplementary material for: Causes of death among patients with hepatocellular carcinoma in United States from 2000 to 2018
Source: Cancer Med. 2023 Apr 21;12(12):13076–85. doi: 10.1002/cam4.5986 (PMC10315789; doi:10.1002/cam4.5986)
Supplement: Supplementary file 13 — Table S10. [file CAM4-12-13076-s014.docx]

| **eTable 10. SMRs for each cause of death following localized HCC diagnosis.** | | | | | | | | | | | |
| --- | --- | --- | --- | --- | --- | --- | --- | --- | --- | --- | --- |
| **Cause of death** | **Deaths by time after diagnosis** | | | | | | | | | **Total deaths** | |
|  | **<2y** | |  | **2-5y** | |  | **>5y** | | |  |  |
|  | **Observed,**  **No.** | **SMR**  **(95% CI)** |  | **Observed,**  **No.** | **SMR**  **(95% CI)** |  | **Observed,**  **No.** | **SMR**  **(95% CI)** |  | **Observed,**  **No.** | **SMR**  **(95% CI)** |
| All | 9539 | 19.60*  (19.32, 19.87) |  | 2823 | 9.90*  (9.65, 10.15) |  | 1255 | 4.37*  (4.20, 4.55) |  | 13617 | 12.90*  (12.75, 13.05) |
| HCC | 7269 | NA |  | 2005 | NA |  | 644 | NA |  | 9918 | NA |
| Other cancers | 424 | 3.72*  (3.48, 3.97) |  | 174 | 2.92*  (2.66, 3.19) |  | 63 | 1.99*  (1.76, 2.24) |  | 661 | 3.05*  (2.90, 3.20) |
| Non-cancer causes | 1846 | 5.75*  (5.57, 5.93) |  | 644 | 3.13*  (2.96, 3.29) |  | 548 | 2.31*  (2.16, 2.47) |  | 3038 | 4.11*  (4.01, 4.21) |
| Cardiovascular diseases | 421 | 2.48*  (2.31, 2.66) |  | 168 | 1.54*  (1.38, 1.72) |  | 161 | 1.47*  (1.30, 1.66) |  | 750 | 1.96*  (1.86, 2.06) |
| Septicemia | 75 | 9.00*  (7.52, 10.69) |  | 20 | 4.91*  (3.60, 6.55) |  | 17 | 3.09*  (2.00, 4.57) |  | 112 | 6.30*  (5.46, 7.24) |
| Pneumonia and Influenza | 28 | 2.68*  (2.03, 3.47) |  | 16 | 1.85*  (1.20, 2.73) |  | 15 | 2.09*  (1.35, 3.09) |  | 59 | 2.29*  (1.88, 2.77) |
| COPD | 57 | 1.83*  (1.49, 2.22) |  | 23 | 1.14  (0.82, 1.54) |  | 27 | 1.59*  (1.18, 2.08) |  | 107 | 1.56*  (1.35, 1.80) |
| Other Infectious and Parasitic Diseases including HIV | 540 | 119.93*  (113.72, 126.39) |  | 159 | 54.41*  (49.21, 60.00) |  | 87 | 26.92*  (22.88, 31.48) |  | 786 | 78.67*  (75.22, 82.24) |
| Diabetes Mellitus | 67 | 3.78*  (3.17, 4.48) |  | 32 | 2.07*  (1.52, 2.74) |  | 26 | 2.17*  (1.58, 2.91) |  | 125 | 2.86*  (2.50, 3.26) |
| Nephritis, Nephrotic Syndrome and Nephrosis | 57 | 4.80*  (3.88, 5.88) |  | 17 | 3.27*  (2.34, 4.43) |  | 35 | 4.51*  (3.34, 5.96) |  | 109 | 4.28*  (3.68, 4.94) |
| Accidents and adverse effects of medications | 62 | 3.58*  (2.97, 4.27) |  | 33 | 3.11*  (2.42, 3.93) |  | 32 | 2.67*  (1.98, 3.51) |  | 127 | 3.21*  (2.82, 3.64) |
| Suicide and Self-Inflicted Injury | 16 | 2.49*  (1.63, 3.65) |  | 7 | 2.06*  (1.13, 3.46) |  | 4 | 1.26  (0.51, 2.59) |  | 27 | 2.06*  (1.52, 2.74) |
| Other | 523 | 7.80*  (7.33, 8.29) |  | 169 | 4.09*  (3.68, 4.54) |  | 144 | 2.57*  (2.33, 2.95) |  | 836 | 5.34*  (5.08, 5.61) |
| **SMR, standard mortality ratio; HCC, hepatocellular carcinoma; COPD,chronic obstructive pulmonary disease; NA, not applicable; CI, confidence interval. * P < 0.05.** | | | | | | | | | | | |
