# Supplementary material for: Causes of death among patients with hepatocellular carcinoma in United States from 2000 to 2018
Source: Cancer Med. 2023 Apr 21;12(12):13076–85. doi: 10.1002/cam4.5986 (PMC10315789; doi:10.1002/cam4.5986)
Supplement: Supplementary file 14 — Table S11. [file CAM4-12-13076-s019.docx]

| **eTable 11. SMRs for each cause of death following regional HCC diagnosis.** | | | | | | | | | | | |
| --- | --- | --- | --- | --- | --- | --- | --- | --- | --- | --- | --- |
| **Cause of death** | **Deaths by time after diagnosis** | | | | | | | | | **Total deaths** | |
|  | **<2y** | |  | **2-5y** | |  | **>5y** | | |  |  |
|  | **Observed,**  **No.** | **SMR**  **(95% CI)** |  | **Observed,**  **No.** | **SMR**  **(95% CI)** |  | **Observed,**  **No.** | **SMR**  **(95% CI)** |  | **Observed,**  **No.** | **SMR**  **(95% CI)** |
| All | 8339 | 51.83*  (51.06, 52.61) |  | 1003 | 16.18*  (15.51, 16.87) |  | 279 | 5.15*  (4.72, 5.61) |  | 9621 | 34.97*  (34.48, 35.45) |
| HCC | 6991 | NA |  | 793 | NA |  | 163 | NA |  | 7947 | NA |
| Other cancers | 472 | 10.52*  (9.55, 11.23) |  | 65 | 4.09*  (3.45, 4.82) |  | 17 | 2.59*  (2.01, 3.29) |  | 554 | 7.59*  (7.15, 8.04) |
| Non-cancer causes | 876 | 9.02*  (8.64, 9.41) |  | 145 | 3.59*  (3.22, 3.99) |  | 99 | 2.25*  (1.92, 2.62) |  | 1120 | 6.51*  (6.26, 6.77) |
| Cardiovascular diseases | 201 | 3.01*  (2.36, 3.78) |  | 24 | 2.12*  (1.31, 3.24) |  | 32 | 1.57  (0.81, 2.74) |  | 257 | 2.54*  (2.08, 3.07) |
| Septicemia | 31 | 13.88*  (10.78, 17.60) |  | 5 | 5.50*  (2.75, 9.84) |  | 4 | 3.33*  (1.08, 7.78) |  | 40 | 10.00*  (7.98, 12.38) |
| Pneumonia and Influenza | 18 | 3.33*  (2.11, 4.99) |  | 5 | 3.52*  (1.69, 6.47) |  | 1 | 1.26  (0.26, 3.67) |  | 24 | 2.96*  (2.08, 4.10) |
| COPD | 25 | 2.70*  (2.01, 3.55) |  | 2 | 0.65  (0.21, 1.52) |  | 5 | 0.99  (0.36, 2.16) |  | 32 | 1.90*  (1.46, 2.44) |
| Other Infectious and Parasitic Diseases including HIV | 244 | 188.90*  (175.99, 202.51) |  | 43 | 64.72*  (52.99, 78.28) |  | 16 | 35.65*  (25.35, 48.74) |  | 303 | 135.40*  (126.88, 144.34) |
| Diabetes Mellitus | 30 | 4.59*  (3.47, 5.96) |  | 6 | 2.15*  (1.07, 3.85) |  | 6 | 2.30*  (1.05, 4.36) |  | 42 | 3.58*  (2.82, 4.48) |
| Nephritis, Nephrotic Syndrome and Nephrosis | 19 | 5.71*  (4.02, 7.87) |  | 1 | 1.88  (0.61, 4.39) |  | 6 | 3.40*  (1.37, 7.01) |  | 26 | 4.38*  (3.24, 5.79) |
| Accidents and adverse effects of medications | 26 | 5.46*  (4.21, 6.95) |  | 8 | 4.09*  (2.50, 6.31) |  | 3 | 1.12  (0.31, 2.87) |  | 37 | 4.37*  (3.51, 5.38) |
| Suicide and Self-Inflicted Injury | 4 | 2.10  (0.91, 4.14) |  | 0 | / |  | 1 | 1.85  (0.22, 6.70) |  | 5 | 1.55  (0.74, 2.85) |
| Other | 278 | 13.66*  (12.59, 14.81) |  | 51 | 5.53*  (4.50, 6.73) |  | 25 | 2.76*  (1.97, 3.76) |  | 354 | 9.65*  (8.97, 10.38) |
| **SMR, standard mortality ratio; HCC, hepatocellular carcinoma; COPD,chronic obstructive pulmonary disease; NA, not applicable; CI, confidence interval. * P < 0.05.** | | | | | | | | | | | |
