# Supplementary material for: Causes of death among patients with hepatocellular carcinoma in United States from 2000 to 2018
Source: Cancer Med. 2023 Apr 21;12(12):13076–85. doi: 10.1002/cam4.5986 (PMC10315789; doi:10.1002/cam4.5986)
Supplement: Supplementary file 15 — Table S12. [file CAM4-12-13076-s016.docx]

| **eTable 12. SMRs for each cause of death following distant HCC diagnosis.** | | | | | | | | | | | |
| --- | --- | --- | --- | --- | --- | --- | --- | --- | --- | --- | --- |
| **Cause of death** | **Deaths by time after diagnosis** | | | | | | | | | **Total deaths** | |
|  | **<2y** | |  | **2-5y** | |  | **>5y** | | |  |  |
|  | **Observed,**  **No.** | **SMR**  **(95% CI)** |  | **Observed,**  **No.** | **SMR**  **(95% CI)** |  | **Observed,**  **No.** | **SMR**  **(95% CI)** |  | **Observed,**  **No.** | **SMR**  **(95% CI)** |
| All | 7275 | 105.81*  (103.90, 107.75) |  | 249 | 22.90*  (20.80, 25.16) |  | 34 | 6.37*  (4.85, 8.22) |  | 7558 | 87.82*  (86.27, 89.39) |
| HCC | 5734 | NA |  | 203 | NA |  | 22 | NA |  | 5959 | NA |
| Other cancers | 1042 | 57.17*  (54.37, 60.09) |  | 27 | 10.29*  (7.56, 13.68) |  | 5 | 4.13*  (1.89, 7.84) |  | 1074 | 47.45*  (45.16, 49.83) |
| Non-cancer causes | 499 | 11.25*  (10.53, 12.01) |  | 19 | 3.19*  (2.32, 4.28) |  | 7 | 2.66*  (1.58, 4.20) |  | 525 | 9.56*  (8.96, 10.18) |
| Cardiovascular diseases | 149 | 5.71*  (4.97, 6.52) |  | 3 | 1.09  (0.44, 2.25) |  | 1 | 0.66  (0.08, 2.38) |  | 153 | 4.75*  (4.15, 5.42) |
| Septicemia | 13 | 13.55*  (8.49, 20.52) |  | 1 | 3.57  (0.09, 19.30) |  | 1 | 14.43*  (1.75, 52.14) |  | 15 | 12.25*  (7.92, 18.08) |
| Pneumonia and Influenza | 7 | 4.09*  (1.96, 7.52) |  | 2 | 6.96*  (1.44, 20.34) |  | 0 | / |  | 9 | 4.22*  (2.24, 7.21) |
| COPD | 18 | 4.60*  (3.08, 6.61) |  | 1 | 1.79  (0.22, 6.46) |  | 0 | 1.88  (0.05, 10.48) |  | 19 | 4.03*  (2.75, 5.68) |
| Other Infectious and Parasitic Diseases including HIV | 121 | 207.98*  (184.08, 234.12) |  | 3 | 48.38*  (23.20, 88.97) |  | 1 | 19.82*  (2.40, 71.60) |  | 125 | 175.99*  (156.19, 197.62) |
| Diabetes Mellitus | 13 | 5.50*  (3.40, 8.40) |  | 3 | 6.21*  (1.69, 15.91) |  | 0 | / |  | 16 | 5.21*  (3.37, 7.70) |
| Nephritis, Nephrotic Syndrome and Nephrosis | 13 | 10.84*  (6.95, 16.13) |  | 1 | 2.58  (0.07, 14.40) |  | 1 | 10.19*  (1.23, 36.80) |  | 15 | 9.65*  (6.36, 14.04) |
| Accidents and adverse effects of medications | 16 | 10.14*  (7.17, 13.91) |  | 0 | 1.57  (0.04, 0.77) |  | 0 | / |  | 16 | 8.28*  (5.89, 11.31) |
| Suicide and Self-Inflicted Injury | 3 | 2.72  (0.56, 7.95) |  | 0 | / |  | 0 | / |  | 3 | 2.92  (0.80, 7.47) |
| Other | 146 | 14.62*  (12.74, 16.70) |  | 5 | 5.19*  (2.84, 8.71) |  | 3 | 4.33*  (1.59, 9.41) |  | 154 | 12.52*  (10.98, 14.22) |
| **SMR, standard mortality ratio; HCC, hepatocellular carcinoma; COPD,chronic obstructive pulmonary disease; NA, not applicable; CI, confidence interval. * P < 0.05.** | | | | | | | | | | | |
