# Supplementary material for: Causes of death among patients with hepatocellular carcinoma in United States from 2000 to 2018
Source: Cancer Med. 2023 Apr 21;12(12):13076–85. doi: 10.1002/cam4.5986 (PMC10315789; doi:10.1002/cam4.5986)
Supplement: Supplementary file 16 — Table S13. [file CAM4-12-13076-s001.docx]

| **eTable 13. SMRs for each cause of death following HCC diagnosis in patients who underwent cancer-directed surgery.** | | | | | | | | | | | |
| --- | --- | --- | --- | --- | --- | --- | --- | --- | --- | --- | --- |
| **Cause of death** | **Deaths by time after diagnosis** | | | | | | | | | **Total deaths** | |
|  | **<2y** | |  | **2-5y** | |  | **>5y** | | |  |  |
|  | **Observed,**  **No.** | **SMR**  **(95% CI)** |  | **Observed,**  **No.** | **SMR**  **(95% CI)** |  | **Observed,**  **No.** | **SMR**  **(95% CI)** |  | **Observed,**  **No.** | **SMR**  **(95% CI)** |
| All | 4071 | 11.80*  (11.52, 12.10) |  | 2147 | 7.82*  (7.57, 8.09) |  | 1221 | 4.07*  (3.89, 4.25) |  | 7439 | 8.00*  (7.85, 8.14) |
| HCC | 2907 | NA |  | 1513 | NA |  | 621 | NA |  | 5041 | NA |
| Other cancers | 204 | 2.47*  (2.22, 2.74) |  | 138 | 2.57*  (2.29, 2.87) |  | 64 | 2.04*  (1.80, 2.30) |  | 406 | 2.36*  (2.21, 2.52) |
| Non-cancer causes | 960 | 3.77*  (3.58, 3.97) |  | 496 | 2.44*  (2.27, 2.62) |  | 536 | 2.19*  (2.04, 2.35) |  | 1992 | 2.83*  (2.73, 2.93) |
| Cardiovascular diseases | 169 | 1.44*  (1.27, 1.63) |  | 109 | 1.16  (0.99, 1.34) |  | 160 | 1.43*  (1.25, 1.63) |  | 438 | 1.35*  (1.25, 1.46) |
| Septicemia | 46 | 8.35*  (6.46, 10.63) |  | 15 | 4.03*  (2.65, 5.86) |  | 16 | 2.85*  (1.76, 4.35) |  | 77 | 5.19*  (4.28, 6.23) |
| Pneumonia and Influenza | 19 | 2.04*  (1.28, 3.09) |  | 12 | 1.49  (0.81, 2.50) |  | 13 | 1.75*  (1.05, 2.73) |  | 44 | 1.77*  (1.33, 2.31) |
| COPD | 21 | 1.12  (0.77, 1.56) |  | 17 | 0.81  (0.50, 1.23) |  | 25 | 1.38  (0.99, 1.87) |  | 63 | 1.12  (0.90, 1.36) |
| Other Infectious and Parasitic Diseases including HIV | 312 | 67.09*  (61.14, 73.47) |  | 131 | 40.41*  (35.29, 46.06) |  | 87 | 26.37*  (22.20, 31.10) |  | 530 | 46.59*  (43.48, 49.87) |
| Diabetes Mellitus | 22 | 2.10*  (1.51, 2.84) |  | 26 | 2.24*  (1.59, 3.08) |  | 26 | 2.16*  (1.54, 2.94) |  | 74 | 2.16*  (1.79, 2.59) |
| Nephritis, Nephrotic Syndrome and Nephrosis | 19 | 2.92*  (1.97, 4.17) |  | 12 | 2.03*  (1.20, 3.21) |  | 33 | 4.14*  (2.97, 5.62) |  | 64 | 3.06*  (2.46, 3.77) |
| Accidents and adverse effects of medications | 33 | 2.43*  (1.79, 3.23) |  | 29 | 2.56*  (1.85, 3.46) |  | 28 | 2.20*  (1.56, 3.02) |  | 90 | 2.40*  (2.00, 2.85) |
| Suicide and Self-Inflicted Injury | 11 | 2.01*  (1.07, 3.44) |  | 2 | 0.57  (0.12, 1.67) |  | 4 | 1.35  (0.54, 2.78) |  | 17 | 1.36  (0.86, 2.04) |
| Other | 308 | 5.80*  (5.25, 6.40) |  | 143 | 3.33*  (2.89, 3.82) |  | 144 | 2.53*  (2.18, 2.92) |  | 595 | 3.89*  (3.63, 4.17) |
| **SMR, standard mortality ratio; HCC, hepatocellular carcinoma; COPD,chronic obstructive pulmonary disease; NA, not applicable; CI, confidence interval. * P < 0.05.** | | | | | | | | | | | |
