# Supplementary material for: Causes of death among patients with hepatocellular carcinoma in United States from 2000 to 2018
Source: Cancer Med. 2023 Apr 21;12(12):13076–85. doi: 10.1002/cam4.5986 (PMC10315789; doi:10.1002/cam4.5986)
Supplement: Supplementary file 17 — Table S14. [file CAM4-12-13076-s013.docx]

| **eTable 14. SMRs for each cause of death following HCC diagnosis in patients who underwent chemotherapy.** | | | | | | | | | | | |
| --- | --- | --- | --- | --- | --- | --- | --- | --- | --- | --- | --- |
| **Cause of death** | **Deaths by time after diagnosis** | | | | | | | | | **Total deaths** | |
|  | **<2y** | |  | **2-5y** | |  | **>5y** | | |  |  |
|  | **Observed,**  **No.** | **SMR**  **(95% CI)** |  | **Observed,**  **No.** | **SMR**  **(95% CI)** |  | **Observed,**  **No.** | **SMR**  **(95% CI)** |  | **Observed,**  **No.** | **SMR**  **(95% CI)** |
| All | 8451 | 30.74*  (30.35, 31.13) |  | 1804 | 9.41*  (9.15, 9.69) |  | 503 | 4.26*  (4.07, 4.45) |  | 10758 | 17.66*  (17.46 17.86) |
| HCC | 6965 | NA |  | 1391 | NA |  | 288 | NA |  | 8644 | NA |
| Other cancers | 605 | 8.34*  (7.94, 8.77) |  | 123 | 2.98*  (2.68, 3.30) |  | 38 | 2.01*  (1.75, 2.29) |  | 766 | 5.12*  (4.91, 5.34) |
| Non-cancer causes | 881 | 6.34*  (6.13, 6.55) |  | 290 | 2.96*  (2.78, 3.15) |  | 177 | 2.32*  (2.16, 2.49) |  | 1348 | 4.32*  (4.20, 4.44) |
| Cardiovascular diseases | 183 | 2.88*  (2.68, 3.09) |  | 49 | 1.57*  (1.38, 1.77) |  | 49 | 1.52*  (1.32, 1.73) |  | 281 | 2.16*  (2.04, 2.28) |
| Septicemia | 35 | 9.69*  (7.97, 11.68) |  | 10 | 5.22*  (3.69, 7.16) |  | 5 | 3.46*  (2.19, 5.19) |  | 50 | 6.77*  (5.79, 7.86) |
| Pneumonia and Influenza | 16 | 2.48*  (1.80, 3.33) |  | 9 | 2.08*  (1.32, 3.12) |  | 7 | 1.48  (0.83, 2.43) |  | 32 | 2.10*  (1.67, 2.61) |
| COPD | 28 | 2.31*  (1.89, 2.81) |  | 8 | 1.21  (0.83, 1.68) |  | 9 | 1.54*  (1.10, 2.09) |  | 45 | 1.79*  (1.54, 2.07) |
| Other Infectious and Parasitic Diseases including HIV | 277 | 132.95*  (125.41, 140.84) |  | 89 | 51.48*  (45.69, 57.80) |  | 30 | 26.07*  (21.65, 31.13) |  | 396 | 82.66*  (78.62, 86.86) |
| Diabetes Mellitus | 24 | 4.30*  (3.56, 5.15) |  | 15 | 2.52*  (1.84, 3.37) |  | 11 | 2.07*  (1.43, 2.89) |  | 50 | 3.19*  (2.76, 3.67) |
| Nephritis, Nephrotic Syndrome and Nephrosis | 15 | 5.92*  (4.78, 7.27) |  | 6 | 2.31*  (1.46, 3.47) |  | 13 | 4.52*  (3.24, 6.13) |  | 34 | 4.51*  (3.83, 5.28) |
| Accidents and adverse effects of medications | 30 | 5.34*  (4.49, 6.31) |  | 13 | 3.10*  (2.31, 4.06) |  | 8 | 2.57*  (1.83, 3.52) |  | 51 | 3.96*  (3.47, 4.51) |
| Suicide and Self-Inflicted Injury | 7 | 3.21*  (2.06, 4.78) |  | 4 | 1.42  (0.57, 2.92) |  | 1 | 1.15*  (0.37, 2.69) |  | 12 | 2.15*  (1.51, 2.97) |
| Other | 266 | 8.51*  (7.96, 9.08) |  | 87 | 3.63*  (3.19, 4.10) |  | 44 | 2.72*  (2.34, 3.15) |  | 397 | 5.53*  (5.23, 5.83) |
| **SMR, standard mortality ratio; HCC, hepatocellular carcinoma; COPD,chronic obstructive pulmonary disease; NA, not applicable; CI, confidence interval. * P < 0.05.** | | | | | | | | | | | |
