# Supplementary material for: Causes of death among patients with hepatocellular carcinoma in United States from 2000 to 2018
Source: Cancer Med. 2023 Apr 21;12(12):13076–85. doi: 10.1002/cam4.5986 (PMC10315789; doi:10.1002/cam4.5986)
Supplement: Supplementary file 18 — Table S15. [file CAM4-12-13076-s006.docx]

| **eTable 15. SMRs for each cause of death following HCC diagnosis in patients who underwent radiation.** | | | | | | | | | | | |
| --- | --- | --- | --- | --- | --- | --- | --- | --- | --- | --- | --- |
| **Cause of death** | **Deaths by time after diagnosis** | | | | | | | | | **Total deaths** | |
|  | **<2y** | |  | **2-5y** | |  | **>5y** | | |  |  |
|  | **Observed,**  **No.** | **SMR**  **(95% CI)** |  | **Observed,**  **No.** | **SMR**  **(95% CI)** |  | **Observed,**  **No.** | **SMR**  **(95% CI)** |  | **Observed,**  **No.** | **SMR**  **(95% CI)** |
| All | 2399 | 29.63*  (28.72, 30.57) |  | 317 | 12.80*  (11.84, 13.82) |  | 46 | 4.70*  (3.84, 5.70) |  | 2762 | 22.80*  (22.15, 23.46) |
| HCC | 1990 | NA |  | 237 | NA |  | 29 | NA |  | 2256 | NA |
| Other cancers | 228 | 9.94*  (8.89, 11.08) |  | 35 | 5.78*  (4.50, 7.30) |  | 2 | 1.59  (0.69, 3.13) |  | 265 | 8.09*  (7.32, 8.92) |
| Non-cancer causes | 181 | 4.10*  (3.71, 4.53) |  | 45 | 2.95*  (2.43, 3.56) |  | 15 | 2.14*  (1.49, 2.98) |  | 241 | 3.60*  (3.30, 3.92) |
| Cardiovascular diseases | 48 | 1.68*  (1.32, 2.11) |  | 12 | 1.58*  (1.04, 2.30) |  | 4 | 0.96  (0.39, 1.98) |  | 64 | 1.58*  (1.30, 1.91) |
| Septicemia | 5 | 7.53*  (4.22, 12.43) |  | 2 | 6.55*  (2.13, 15.29) |  | 0 | / |  | 7 | 6.50*  (3.97, 10.04) |
| Pneumonia and Influenza | 2 | 2.55  (0.98, 5.04) |  | 1 | 2.61  (0.54, 7.63) |  | 0 | / |  | 3 | 2.20*  (1.05, 4.05) |
| COPD | 5 | 1.13  (0.52, 2.15) |  | 1 | 1.32  (0.36, 3.39) |  | 1 | 2.97  (0.81, 7.61) |  | 7 | 1.38  (0.80, 2.21) |
| Other Infectious and Parasitic Diseases including HIV | 42 | 88.18*  (73.56, 104.84) |  | 5 | 46.52*  (29.80, 69.21) |  | 2 | 33.60*  (13.51, 69.23) |  | 49 | 73.07*  (62.16, 85.36) |
| Diabetes Mellitus | 6 | 2.12*  (1.02, 3.90) |  | 4 | 2.79  (0.91, 6.52) |  | 0 | / |  | 10 | 2.20*  (1.26, 3.58) |
| Nephritis, Nephrotic Syndrome and Nephrosis | 6 | 2.45  (0.98, 5.04) |  | 1 | 2.61  (0.54, 7.63) |  | 0 | / |  | 7 | 2.20*  (1.05, 4.05) |
| Accidents and adverse effects of medications | 8 | 4.10*  (2.47, 6.40) |  | 2 | 4.09*  (1.65, 8.43) |  | 1 | 1.42  (0.04, 7.92) |  | 11 | 3.83*  (2.52, 5.57) |
| Suicide and Self-Inflicted Injury | 1 | 1.47  (0.18, 5.31) |  | 0 | / |  | 1 | 5.75  (0.15, 32.01) |  | 2 | 2.50  (0.81, 5.83) |
| Other | 58 | 5.79*  (4.76, 6.97) |  | 17 | 3.63*  (2.41, 5.25) |  | 6 | 2.89*  (1.39, 5.32) |  | 81 | 4.91*  (4.15, 5.77) |
| **SMR, standard mortality ratio; HCC, hepatocellular carcinoma; COPD,chronic obstructive pulmonary disease; NA, not applicable; CI, confidence interval. * P < 0.05.** | | | | | | | | | | | |
