# Supplementary material for: Causes of death among patients with hepatocellular carcinoma in United States from 2000 to 2018
Source: Cancer Med. 2023 Apr 21;12(12):13076–85. doi: 10.1002/cam4.5986 (PMC10315789; doi:10.1002/cam4.5986)
Supplement: Supplementary file 19 — Legends [file CAM4-12-13076-s015.docx]

**eFigure 1**. Proportions of deaths due to non-cancer causes by each calendar year.

**eFigure 2**. Standard mortality ratios (SMRs) for the common causes of non-cancer death were characterized after diagnosis, binned by patient age groups.

**eFigure 3**. Standard mortality ratios (SMRs) for the common causes of non-cancer death were characterized after diagnosis, binned by time periods.
